# PhaMMseqs: a new pipeline for constructing phage gene phamilies using MMseqs2

**DOI:** 10.1093/g3journal/jkac233

**Published:** 2022-09-26

**Authors:** Christian H Gauthier, Steven G Cresawn, Graham F Hatfull

**Affiliations:** Department of Biological Sciences, University of Pittsburgh, Pittsburgh, PA 15260, USA; Department of Biology, James Madison University, Harrisonburg, VA 22807, USA; Department of Biological Sciences, University of Pittsburgh, Pittsburgh, PA 15260, USA

**Keywords:** phage genomics, bacteriophages, comparative genomics

## Abstract

The diversity and mosaic architecture of phage genomes present challenges for whole-genome phylogenies and comparative genomics. There are no universally conserved core genes, ∼70% of phage genes are of unknown function, and phage genomes are replete with small (<500 bp) open reading frames. Assembling sequence-related genes into “phamilies” (“phams”) based on amino acid sequence similarity simplifies comparative phage genomics and facilitates representations of phage genome mosaicism. With the rapid and substantial increase in the numbers of sequenced phage genomes, computationally efficient pham assembly is needed, together with strategies for including newly sequenced phage genomes. Here, we describe the Python package PhaMMseqs, which uses MMseqs2 for pham assembly, and we evaluate the key parameters for optimal pham assembly of sequence- and functionally related proteins. PhaMMseqs runs efficiently with only modest hardware requirements and integrates with the pdm_utils package for simple genome entry and export of datasets for evolutionary analyses and phage genome map construction.

## Introduction

Bacteriophages (phages) are an enormous, genetically diverse population whose evolution likely spans billions of years (Hendrix *et al.* 1999; Hendrix 2002). A hallmark genomic feature is their pervasive mosaicism with related genes situated in different genomic contexts in otherwise unrelated phages (Pedulla *et al.* 2003). This confounds simple phylogenetic representations, and phage diversity is sufficiently great that there are no universally conserved “core” genes (Hatfull and Hendrix 2011); phages do not have the equivalent of bacterial 16S rRNA genes. Because horizontal exchange events giving rise to genomic mosaicism are prevalent, they are key factors in phage evolution together with ongoing variation at the nucleotide sequence level (Lima-Mendez *et al.* 2008). Comparison of nucleotide divergence and rates of gene exchange reveals evolutionary patterns that differ with different hosts and phage lifestyles (Mavrich and Hatfull 2017). However, this requires an assortment of phage genes into related groups, or “phamilies.”

Assortment of phage genes into phamilies (phams) was initially described using Phamerator, which assembled phams (using amino acid sequences) with specific BLASTP and CLUSTALW parameters (Cresawn *et al.* 2011). The extant target dataset was modest—80 mycobacteriophage genomes encompassing ∼800 genes—and was computationally manageable. The output of the pham dataset was useful for many downstream analyses and representations, including comparative genome maps using the Phamerator display functions (Cresawn *et al.* 2011). As the number of phage genomes rapidly increased, the computational demands exceeded the capacity of BLASTP and CLUSTALW, and a revised Pham building system was developed using kClust and associated programs (Pope *et al.* 2015). However, kClust (Hauser *et al.* 2013) was soon deprecated and replaced by the more powerful MMseqs2 package (Steinegger and Soding 2017).

Here, we describe PhaMMseqs, a new pipeline for Pham assembly, using MMseqs2. Phage gene sequences are first translated into amino acid sequences, and PhaMMseqs uses MMseqs2 to first derive sequence profiles, followed by profile-sequence clustering to merge phams containing more remote homologs; Clustal Omega (Sievers *et al.* 2011) is used to construct pham multiple sequence alignments (MSAs). PhaMMseqs can readily assemble Phams from large genome datasets (>500,000 genes) on modest hardware and can be used in combination with database management utilities like pdm_utils (Mavrich *et al.* 2021) for efficiently updating phams as the genome annotation landscape changes.

## Materials and methods

### PhaMMseqs programming

PhaMMseqs was written in Python, is compatible with Python versions 3.7 and above, and uses Biopython (Cock *et al.* 2009) to parse GenBank flat files and MMseqs2 (Steinegger and Soding 2017) for pham assembly. Clustal Omega (Sievers *et al.* 2011) is used optionally to compute MSAs. The PhaMMSeqs package is available at PyPI (https://pypi.org/project/phammseqs/) or GitHub (https://github.com/chg60/PhaMMseqs.git). MMseqs2 and Clustal Omega must be installed separately, using a package manager, such as Anaconda or compiled manually; PhaMMseqs installation and usage instructions are available in the GitHub repository. PhaMMseqs has also been incorporated into the *pdm_utils* package (Mavrich *et al.* 2021) for creating and maintaining phage genome databases, replacing its “phamerate” pipeline (i.e. the prior system for pham assembly).

### Computing global alignments

Phage gene sequences were translated into amino acid sequences using a standard genetic code (11: Bacterial, Archaeal and Plant Plastid) and Needleman–Wunsch global amino acid sequence alignments were computed using the PARASAIL library (Daily 2016). Alignment scores were calculated between sequence pairs using the BLOSUM62 substitution matrix (Henikoff and Henikoff 1992) with gap-opening and extension penalties of −11 and −1, respectively. Alignment statistics (identity, similarity, gap percentage, and length-normalized bitscore) for each sequence pair were cached to avoid recomputing alignments.

### Identifying false-positive pham members

A gene present within a pham that should not be (i.e. a nonhomolog) is considered a false positive. To identify false positives, pairwise global alignments were first computed within each pham. A sequence was counted as a false positive if either of 2 conditions were met: (1) the gene’s length is less than 60% of the length of the longest pham member or (2) the gene’s best pairwise alignment within the pham is not significantly better than an alignment between unrelated gene pairs (length-normalized score threshold: 0.5 half-bits per column). The former identifies any gene whose homology is evidently nonglobal in scope, while the latter finds genes with dubious homology to the rest of the pham (see the* Methods* in *Supplementary Materials* and Supplementary Fig. 1 for the establishment of these thresholds).

### Identifying false-negative pham members

A gene expected to be present in a pham but which is absent is considered a false negative. To identify false-negative pham members, pairwise global alignments were computed within each pham, and the gene with the highest average similarity to all other genes was selected as the pham representative. BLASTP was used to identify genes in the complete dataset that are likely globally homologous to the pham representative (*E*-value 0.001, query coverage at least 60%, query length no less than 60% of subject length). Any such genes identified by BLASTP but not present in the pham were counted as false negatives.

### Determining and validating pham consensus functions

For every pham, we identified the set of unique functional annotations ascribed to members of the pham and counted how many times each was used. Counts were manually adjusted to account for labeling inconsistencies (e.g. “terminase, large subunit,” “TerL,” “large terminase” all refer to the same function) and typographic errors within individual genome annotations. The most common function in each pham was used as its consensus function. For validation of consensus functions, pham MSAs were submitted to the Max Planck Institute’s HHpred server (https://toolkit.tuebingen.mpg.de/tools/hhpred), with the following 4 structural/domain databases selected for search: PDB_mmCIF70, PDB_mmCIF30, UniProt-SwissProt-viral70, and NCBI_ConservedDomains(CD). Top hits were manually evaluated for agreement with the annotated functions.

## Results

### PhaMMseqs workflow

**Fig. 1.**
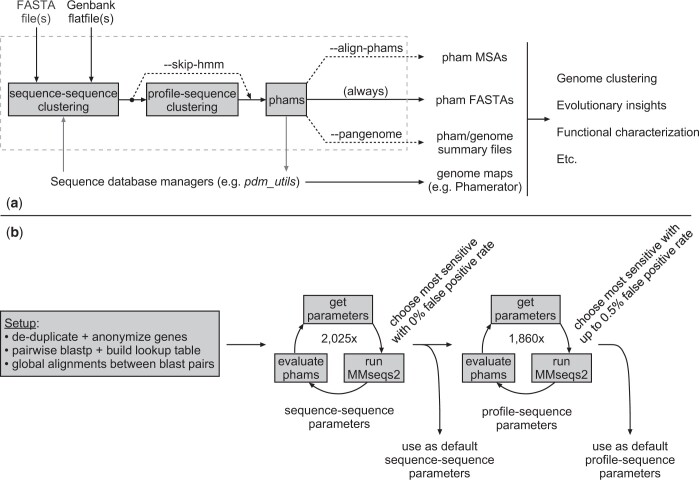
Pipelines for pham assembly and evaluation. a) Input FASTA or GenBank files are parsed for amino-acid sequences, which are clustered using sequence–sequence alignments by PhaMMseqs; these are then further clustered using profile–sequence alignments. The command line argument “–skip-hmm” allows users to bypass profile–sequence clustering if desired. The resulting phams are written to FASTA multiple sequence files, and pham MSAs optionally computed with Clustal Omega if using the “–align-phams” argument. If each input file contains genes from a single genome, the “–pangenome” argument will produce Roary-inspired pham/genome summary files. The programmatic interface allows scripts or larger suites of tools (including the *pdm_utils* package for maintaining Phamerator databases) to directly interact with the pham assembly workflow without producing any unwanted output files. PhaMMseqs’s output files can be used for numerous downstream analyses. b) PhaMMseqs training workflow. Initial setup steps were implemented to avoid computationally expensive training tasks, and then several thousand sequence–sequence parameter sets were run to identify those with greatest sensitivity and minimal false positives. These parameters were used to build pham profiles, which were used with thousands of profile–sequence parameter sets to identify those assembling phams with greatest sensitivity and a false positive rate below 0.5%. The most sensitive parameter sets in both stages were selected as the default MMseqs2 parameters used by PhaMMseqs.

Understanding bacteriophage evolution requires comparative analysis of their gene organization and gene content, together with insights into gene function. The genomes are characteristically mosaic and the relationships span considerable timespans, with many genes of common origins no longer sharing recognizable nucleotide sequence similarity. A central component of this analysis is thus to arrange phage genes into groups (phamilies or phams) based on shared amino acid sequences, and the primary function of PhaMMseqs is to perform pham assembly, which it does by interfacing with the sequence–sequence and profile–sequence clustering pipelines of MMseqs2. The program workflow (Fig. 1a) proceeds as follows. Protein-coding sequences from one or more input files in FASTA or GenBank flat file format are collected and clustered with MMseqs2 according to user-specified or default parameters. Optionally, gene phamilies containing remote homologs can then be merged by a profile–sequence clustering step. Each resultant pham is written to an FASTA multiple sequence file, and pham MSAs can be optionally computed using a local installation of Clustal Omega. If each input file contains the genes from a single genome, 3 pangenome-style analyses can also be performed (Fig. 1a). The first scores the degree of conservation of each pham among input genomes, labeling each pham as containing “core,” “soft-core,” “shell,” or “cloud” genes according to the percentage of genomes (>99%, >95%, >15%, or >0%, respectively) encoding a gene assorted into that pham. The second follows Roary (Page *et al.* 2015) and creates a comma-separated value file with pham-wise summary statistics and a presence–absence matrix indicating which (if any) genes from each genome are assorted into each pham. The last creates a tab-separated value file mapping each genome to the phams its genes have been assorted into and is useful for other tools that perform genome clustering based on shared phams.

### MMseqs2 parameter selection and value optimization

The performance of MMseqs2 can be carefully tuned by specifying values for any of dozens of parameters, some of which have more significant and/or intuitive impacts on clustering than others. We focused on 6 of these parameters that principally define how MMseqs2 finds homologous gene pairs and how it interprets the homology network into gene clusters: minimum sequence identity (–min-seq-id), coverage (-c), *E*-value (-e), sensitivity (-s; used to allow nonidentical k-mer matches to seed alignments), the number of cluster steps (–cluster-steps/–num-iterations), and the clustering algorithm itself (–cluster-mode). For each of these parameters, the value that PhaMMseqs uses by default was carefully optimized to maximize the sensitivity in grouping remote global homologs while avoiding domain-chaining (Joseph and Durand 2009) and other false positives. Domain chaining is the act of grouping globally dissimilar proteins based on local homology (i.e. a shared domain) and reduces the effectiveness of phams as tools for exploring gene function or genome mosaicism. Our training dataset comprised 89,208 nonredundant amino-acid sequences (181,625 including identical sequences found in multiple genomes) derived from the annotations of 1,885 phages infecting 13 genera of Actinobacteria (Supplementary Table 1), retrieved from PhagesDB (Russell and Hatfull 2017).

Parameter training was done in 2 stages by a pipeline we created to run PhaMMseqs across ranges of values for each parameter and evaluate the resultant phams for likely false positives and false negatives (Fig. 1b). In the first stage (Table 1), we evaluated 2,025 parameter sets with the goal of building inclusive yet highly specific (i.e. avoiding non homologous members) phams that could be used as high-quality sequence profiles for more remote homology detection. For this stage, any parameter sets producing at least 1 pham with false positives were excluded, thus reducing noise that compromises the ability of a profile to find true remote homologs and increases the likelihood of accumulating false positives during profile-based clustering. In the second stage (Table 2), we evaluated 1,890 profile-sequence clustering parameter sets using profiles built from the best first-stage parameters, and with the goals of maximizing sensitivity (i.e. maximal inclusion of homologs) and maintaining a low percentage of genes identified as false positives in their respective phams.

**Table 1.** Parameters used in sequence–sequence clustering grid search.

Parameter name	Values	Brief description
—cluster-mode	0, 1, 2	Clustering algorithm to use^a^
—cluster-steps	1, 2, 3	Number of cascaded clustering steps to do^b^
−s	1, 4, 7	Sensitivity^c^
—min-seq-id	0.5, 0.45, 0.4, 0.35, 0.3	Percent identity threshold
−c	0.9, 0.85, 0.8, 0.75, 0.7	Coverage threshold
−e	1e−10, 1e−05, 1e−3	*E*-value threshold

a Different algorithms are available to interpret the graph of pairwise edges into clusters. 0 = set cover, 1 = single-linkage (like blastclust), 2 = greedy-incremental (like CD-HIT). Details are in MMseqs2 manual.

b Performs clustering with strict parameters, then incrementally merges clusters by relaxing parameters down to the selected ones in these many steps.

c Higher sensitivity values allow less similar k-mers to count as matches that can seed an alignment.

**Table 2.** Parameters used in profile–sequence clustering grid search.

Parameter name	Values	Brief description
—cluster-mode	0, 1, 2	Clustering algorithm to use^a^
—num-iterations	1, 2, 3	Number of iterations to do^b^
−s	1, 4, 7	Sensitivity^c^
—min-seq-id	0.35, 0.3, 0.25, 0.2, 0.15	Percent identity threshold
−c/—cov	0.85, 0.8, 0.75, 0.7, 0.65, 0.6, 0.55	Coverage threshold
−e/—e-profile	1e−05, 1e−3	*E*-value threshold

a Different algorithms are available to interpret the graph of pairwise edges into clusters. 0 = set cover, 1 = single-linkage (like blastclust), 2 = greedy-incremental (like CD-HIT). Details are in MMseqs2 manual.

b Performs this many PSI-BLAST-like iterations of finding homologs and updating profiles before clustering.

c Higher sensitivity values allow less similar k-mers to count as matches that can seed an alignment.

### Sequence–sequence parameter optimization

**Fig. 2.**
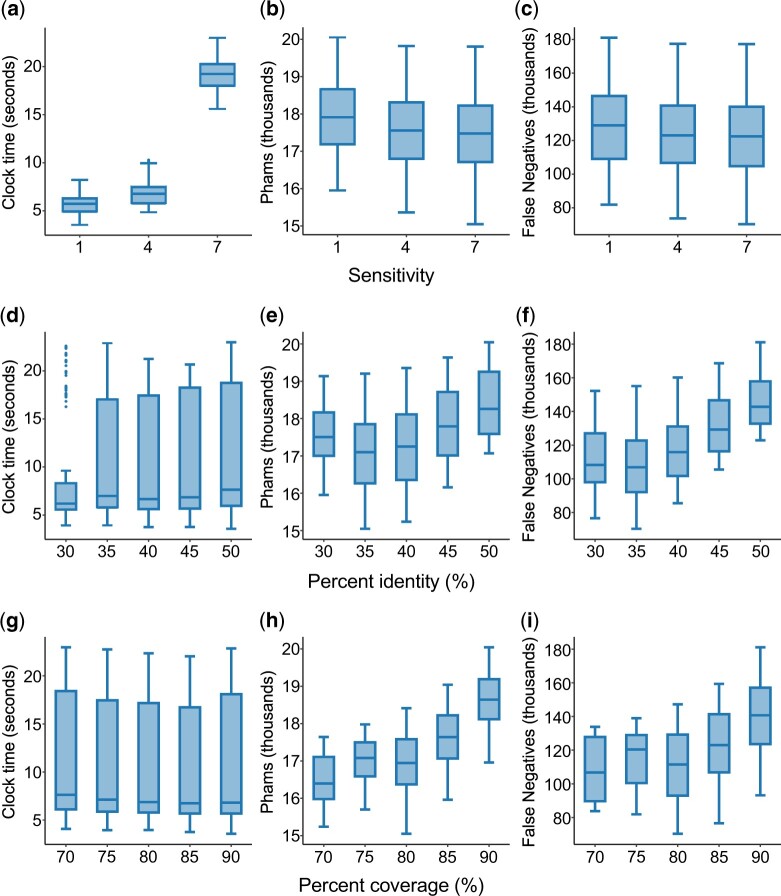
The impact of sensitivity, identity, and coverage on sequence–sequence clustering with MMseqs2. The runtime (in seconds), number of phams, and number of false negatives were plotted for each value of sensitivity (a–c), percent identity (d–f), and percent coverage (g–i) tested (see *Materials and Methods* for identification of false negatives). Sensitivity (-s) is used by MMseqs2 to scale the score threshold required to treat similar k-mers as match-states that can seed an alignment; higher values reduce the score threshold, theoretically resulting in the alignment and evaluation of less similar protein pairs. Percent identity (–min-seq-id) defines how similar a protein must be to a cluster representative in order to join that cluster, with lower values allowing less similar proteins to join a cluster. Percent coverage (-c) defines the proportion of query/target sequences that must be covered by an alignment in order for the proteins to cluster together. Parameter sets that produced any phams with false positives were omitted from this analysis.

Of the 2,025 possible combinations of values given in Table 1, only 1,099 produced phams containing no likely false positives. Of these, we selected the parameter set with the fewest false negatives as the default for sequence–sequence clustering. Analysis of these false-positive-free parameter sets was helpful for understanding how MMseqs2 performs with these datasets. The choice of the MMseqs2 parameter described as “sensitivity” strongly influences runtime (Fig. 2a), with runtime increasing superlinearly with sensitivity. Increasing MMseqs2 sensitivity also tends to reduce the number of phams (Fig. 2b) and false negatives (Fig. 2c), both indications that homologous sequences are being joined into phamilies. Percent identity and coverage thresholds have little impact on runtime (Fig. 2, d and g), but are important determinants of MMseqs2 sensitivity, with lower values for both parameters yielding significantly reduced number of false negatives, and likewise reduced number of phams (Fig. 2, e, f and h, i). Intuitively, these effects are likely to be additive, such that reducing percent identity and coverage thresholds simultaneously results in fewer phams and false negatives than lowering either threshold alone but may also result in introduction of false positives.

**Fig. 3.**
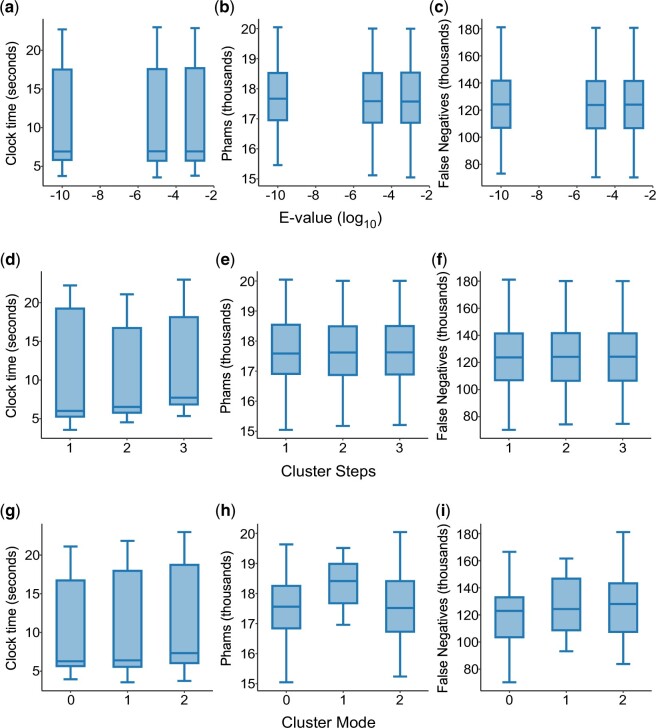
The impact of *E*-value, cluster steps, and cluster mode on sequence–sequence clustering with MMseqs2. The runtime (in seconds), number of phams, and number of false negatives were plotted for each value of *E*-value (a–c), cluster steps (d–f), and cluster mode (g–i) tested (see *Materials and Methods* for identification of false negatives). *E*-value (-e) indicates the statistical significance of a particular alignment, accounting for the size and composition of both the database and the aligned proteins; smaller values are more significant. Cluster steps determine how many relaxing iterations MMseqs2 should perform before clustering at the indicated stringency. Cluster mode (values are not ordinal) selects which algorithm MMseqs2 uses to interpret the homology network into clusters: 0 = set cover, 1 = single-linkage, 2 = greedy-incremental (see MMseqs2 manual for details). Parameter sets that produced any phams with false positives were omitted from this analysis.

Within the tested ranges of values, the *E*-value threshold and number of cluster steps appear not to significantly alter runtime, number of phams, or the number of false negatives (Fig. 3, a–f). The choice of cluster mode (clustering algorithm) has minimal impact on runtime (Fig. 3g) but is clearly important, because the majority (80%) of parameter sets using cluster mode 1 (single-linkage clustering) have at least 1 false-positive member, limiting its utility to much higher percent identity and coverage thresholds than can be used with the other 2 cluster modes. Cluster mode 2, which is similar to the CD-HIT algorithm (Li and Godzik 2006), cluster genes in descending order of length, and appears to be the least sensitive, consistently producing more phams with more false negatives. Of the available cluster modes, we find that cluster mode 0 (“greedy incremental”; clusters genes in descending order of connectivity to other genes) makes the best tradeoff between sensitivity and false positivity.

### Profile–sequence parameter optimization

**Fig. 4.**
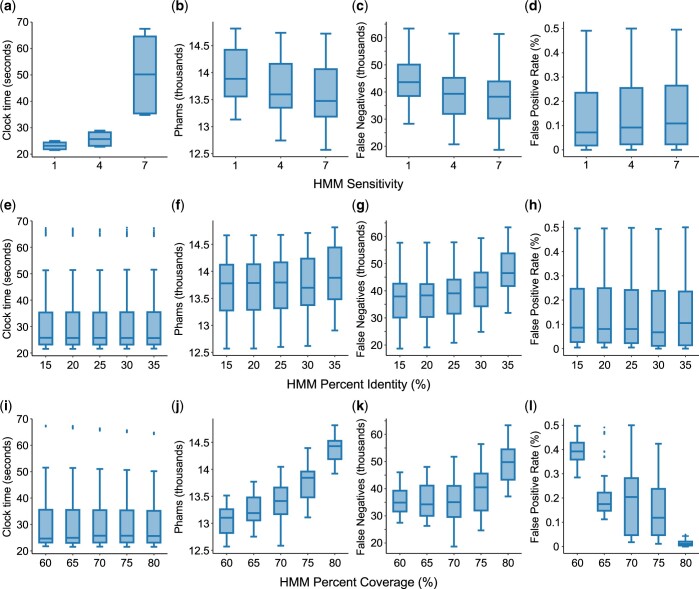
The impact of sensitivity, identity, and coverage on profile–sequence clustering with MMseqs2. The runtime (in seconds), number of phams, number of false negatives, and false positive rate were plotted for each value of sensitivity (a–d), percent identity (e–h), and percent coverage (i–l) tested. False-positive rate was calculated as the number of genes determined to be false positives in their respective phams divided by the number of genes being clustered (see *Materials and Methods* for identification of false positives and negatives). Sensitivity (-s), percent identity (–min-seq-id), and percent coverage (-c) are as described in Fig. 2. Parameter sets producing false positives above a rate of 0.5% were omitted from this analysis.

Of the 1,890 possible combinations of values given in Table 2, only 902 assembled phams with an overall false positive rate below an arbitrarily chosen 0.5%. Analysis of these parameter sets largely recapitulates the trends observed during sequence–sequence clustering. Increasing values of sensitivity resulted in significantly increased runtimes, modest decreases in the number of phams and false negatives, and a slight increase in the number of false positives observed (Fig. 4, a–d). Percent identity has no significant impact on runtime or the incidence of false positives but is positively correlated with the number of phams and false negatives (Fig. 4, e–h). Coverage likewise has no discernable impact on overall runtime but plays a very strong role in the number of observed phams, false negatives, and false positives (Fig. 4, i–l).

**Fig. 5.**
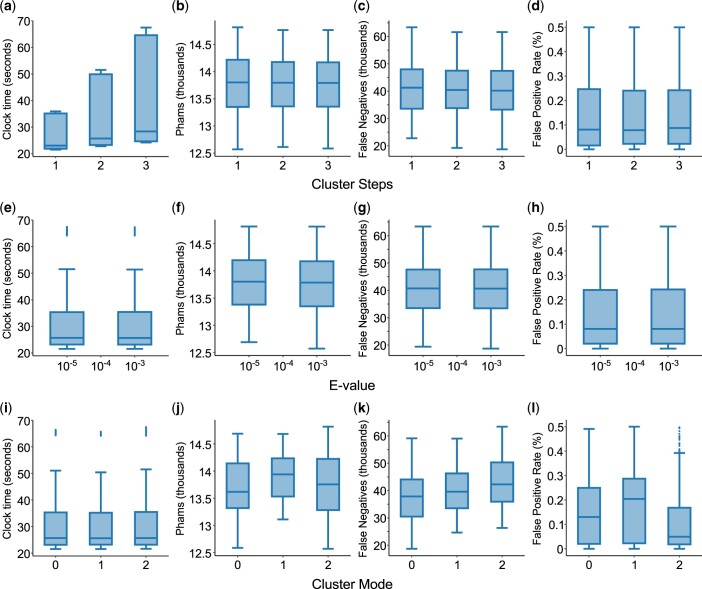
The impact of *E*-value, cluster steps, and cluster mode on sequence–sequence clustering with MMseqs2. The runtime (in seconds), number of phams, number of false negatives, and false-positive rate were plotted for each value of *E*-value (a–d), cluster steps (e–h), and cluster mode (i–l) tested. False-positive rate was calculated as the number of genes determined to be false positives in their respective phams divided by the number of genes being clustered (see *Materials and Methods* for identification of false positives and negatives). *E*-value (-e), cluster steps, and cluster mode are as described in Fig. 3. Parameter sets producing false positives above a rate of 0.5% were omitted from this analysis

As was expected but not observed during sequence–sequence clustering, the number of cluster steps did significantly impact runtime in profile–sequence clustering, with more steps resulting in longer runtimes (Fig. 5a). This is likely due to the cost of having to update profiles with each added step. No other significant effects were observed related to the number of cluster steps or the choice of *E*-value threshold (Fig. 5, b–h). As was observed in sequence–sequence clustering, the single linkage algorithm used in cluster mode 1 has an elevated false-positive rate (Fig. 5, i–l) that reduces its utility at low identity or coverage thresholds. Overall, cluster mode 2 appears less sensitive than the others, reflected in its reduced incidence of false positives and larger numbers of phams and false negatives. As in sequence–sequence clustering, we find cluster mode 0 to have the best overall performance.

### Scalability of optimized parameters

Using PhaMMseqs iteratively with the optimized parameters, we can assemble a redundant set of 181,625 training genes (which includes redundant gene copies excluded from the nonredundant training set) into 17,248 phams with a mean size of 10.53 genes in ∼3 min on a 2020 MacBook Pro with an 8-core M1 processor. The largest pham contains 946 genes, and there are 7,644 (44.3%) phams with only 1 gene (orphams). To test the scalability of the optimized MMseqs2 parameters, we downloaded all 4,380 complete phage annotations available in RefSeq (accessed 2021 August 29). The RefSeq (O’Leary *et al.* 2016) phages comprise representative and reference genomes from each “species” of sequenced phage, thus these genomes are minimally redundant; these phages jointly encode 465,399 genes (345,410 nonredundant). Using the same device as before, these genes assemble into 90,644 phams (∼5.13 genes/pham) in roughly 22 min; the largest pham has 775 genes, and there are 50,804 (56.0%) orphams. The increase in runtime and number of phams and orphams are consistent with the substantial increase in diversity in RefSeq phages compared to the Actinobacteriophages initially trained on; nonetheless, the runtime is clearly not prohibitive even with readily available computational resources.

### Phams produced using optimized parameters are functionally consistent

The default PhaMMseqs parameters were chosen in a manner that theoretically builds phams that are functionally consistent. We analyzed the 20 largest phams from the training and RefSeq datasets to determine the validity of this, as these typically are functionally well-defined and are vulnerable to the potential inclusion of false positives. For the training dataset, the 20 largest phams (Table 3) contain a total of 11,198 genes, or roughly ∼6.2% of all genes. Most of these phams have a clear consensus function annotated; those that do not typically have a weak consensus function and a significant population of genes designated as “hypothetical protein.” MSAs for these 20 phams were manually inspected for signs of domain chaining or other obvious issues, as well as submitted to the MPI HHpred server (Zimmermann *et al.* 2018) to validate the consensus function. No instances of domain chaining were observed, and we were encouraged to see 1 pham (rank 5 by size—terminase, large subunit) that includes both intein-containing and intein-free homologs, because inteins are not uncommon in phage genomes and can interrupt pham assembly (Kelley *et al.* 2016). In addition, the best HHpred hits for each pham were congruent with the consensus annotated function. Extending this analysis to the top 50 largest phams is consistent with these conclusions (data not shown).

**Table 3.** Consensus functions of the 20 largest phams in 1,885 Actinobacteriophages.

Rank	No. of genes in pham	Consensus function^a^	Consensus proportion^b^ (%)
1	946	Minor tail protein	92.3
2	932	Lysin B	95.9
3	689	DNA polymerase I	95.6
4	687	Portal	96.7
5	653	Terminase, large subunit	96.5
6	550	Major capsid protein	96.9
7	548	DNA helicase	93.8
8	540	DNA primase	94.1
9	499	Endonuclease VII	93.0
10	499	Terminase, large subunit	97.2
11	498	Hypothetical protein	63.5^c^
12	494	Hypothetical protein	63.3^d^
13	493	Minor tail protein	91.9
14	474	Tail assembly chaperone	96.4
15	463	Minor tail protein	62.0^e^
16	452	Immunity repressor	87.6
17	446	Head-to-tail connector	58.5^e^
18	445	Head-to-tail connector	54.8^e^
19	445	Hypothetical protein	97.8
20	445	Scaffolding protein	94.6

a Consensus function annotated for members of the pham, manually corrected for synonymous labels and typographic errors.

b Percentage of genes in the pham that have been annotated as some variant of the consensus function.

c Most remaining genes are designated as minor tail protein.

d Most remaining genes are designated as head-to-tail connector.

e Most remaining genes are designated as hypothetical protein.

Commensurate with the increased diversity of the RefSeq dataset, the 20 largest phams (Table 4) contain fewer genes both absolutely and proportionally to the size of the dataset (9,637 genes, ∼2.1% of genes). Again, most of these have clear consensus functions, and just 3 phams have a weak consensus diluted by a significant population of “hypothetical protein” or other nonspecific labels. Pham MSAs reveal no evidence of domain chaining, and the best HHpred hits recapitulate the consensus annotated functions for these phams.

**Table 4.** Consensus functions of the 20 largest phams in 4,380 RefSeq phages.

Rank	Number of genes	Consensus function^a^	Consensus proportion^b^ (%)
1	775	Minor tail protein	91.4
2	682	HNH endonuclease	78.1^c^
3	620	Portal	96.0
4	604	Lysin B	95.2
5	541	Ribonucleotide reductase	94.8
6	541	Recombination endonuclease	89.5
7	503	Lysozyme	93.4
8	485	Terminase, large subunit	95.1
9	479	DNA polymerase I	95.2
10	435	Ribonucleotide reductase	95.9
11	416	UvsW-like helicase	92.8
12	413	Terminase, large subunit	95.9
13	409	DNA helicase	78.7^c^
14	401	DNA primase/helicase	88.5
15	396	Terminase, large subunit	95.0
16	394	dNMP kinase	78.9^c^
17	391	Tail tube protein	88.7
18	387	DnaB-like dsDNA helicase	92.8
19	384	Major capsid protein	93.8
20	381	Clamp loader	88.7

a Consensus function annotated for members of the pham, manually corrected for synonymous labels and typographic errors.

b Percentage of genes in the pham that have been annotated as some variant of the consensus function.

c Most remaining genes are designated as hypothetical proteins or have other nonspecific labels.

## Discussion

Pham assembly is broadly useful for phage comparative genomics, and central to phage database resources used in integrated research-education programs such as PHIRE and SEA-PHAGES (Jordan *et al.* 2014; Hanauer *et al.* 2017; Hatfull 2021). With rapid increases in the number of genomes available for analysis, rapid sequence comparison algorithms are needed, and we demonstrate here that MMseqs2 can be effectively used for pham assembly, both in terms of computational speed and capacity as well as the overall quality of assembled phams. Rapid and efficient pham assembly also facilitates comparative phage genomics including both within closely related lineages, and more broadly among more distantly related phages. We have evaluated PhaMMseqs on phage genomes, but it can be readily used with predicted prophages once extracted from bacterial genome sequences.

Several other systems for organizing phage genes have been described, including ACLAME (Leplae *et al.* 2004), POG (Liu *et al.* 2006; Kristensen *et al.* 2011, 2013), and pVOG (Grazziotin *et al.* 2017). However, some of these resources have not been regularly updated, compromising their overall utility. By incorporating PhaMMseqs into pdm_utils (Mavrich *et al.* 2021), a suite of pipelines for managing phage datasets in different databases, rebuilding phams with newly sequenced genomes should be much simplified. PhaMMseqs also provides a much simpler interface for iterative pham assembly than deploying multiple steps directly with MMseqs2.

Bacteriophage genomes have complex relationships, being pervasively mosaic, highly diverse, and replete with genes of unknown function. Moreover, the number of sequenced phage genomes is small relative to the extant population, and many genomes currently have no sequenced close relatives, and there are many genes without homologs in other phages (i.e. orphams). There are several features of phage genomes that can present challenges to pham assembly including the abundance of small genes, the presence of inteins within genes, and intragene mosaicism leading to multidomain proteins with complex evolutionary histories. Thus, even when optimized, pham assembly is expected to be imperfect, reflected by false-positive inclusion or false-negative rejection of genes into phamilies. In a few instances, we have observed some “pham-instability,” where a single set of clearly homologous genes are split into 2 phams. This is rarely observed with repeated assembles on the same hardware but can emerge with identical runs on different hardware (e.g. a MacBook Pro with M1 CPU vs a server with AMD Opteron 6376 dual CPU). We estimate such phams are rare (<0.1%) and it is unclear which if any software innovations may help to eliminate these. Nonetheless, PhaMMseqs provides a rapid and efficient method for pham assembly with broad utility in phage genomics.

## Supplementary Material

jkac233_Supplementary_Data

## Data Availability

The PhaMMseqs package is available at PyPI (https://pypi.org/project/phammseqs/) and GitHub (https://github.com/chg60/PhaMMseqs.git). Supplemental material is available at G3 online.
