# Supplementary material for: PhaMMseqs: a new pipeline for constructing phage gene phamilies using MMseqs2
Source: G3 (Bethesda). 2022 Sep 26;12(11):jkac233. doi: 10.1093/g3journal/jkac233 (PMC9635663; doi:10.1093/g3journal/jkac233)
Supplement: jkac233_Supplementary_Data [file jkac233_supplementary_data.pdf]

SUPPLEMENTARY MATERIALS

METHODS

Identifying intein-containing genes

Intein-containing genes were identified in the 89,208 non-redundant training genes by querying them with RPSBLAST (Camacho et al. 2009) against the NCBI's Conserved Domain Database (Marchler-Bauer et al. 2015) to find hits to any of the intein domains in that dataset: cd00081, cd00283, COG1372, pfam13403, pfam14623, pfam14890, TIGR01443, TIGR01445, smart00305, or smart00306. Hits with E-value better than 0.001 were retained and intein coordinates were recorded for each query as the minimum spanning coordinate range that covers all intein domain hits found within a single gene.

Establishing similarity and coverage thresholds for pham evaluation

To choose a similarity threshold for pham evaluation, training genes lacking significant (E-value <= 0.001, no identity/coverage thresholds) non-self hits to the remaining training genes were globally aligned to each other in a pairwise fashion to mimic the alignment of random (unrelated) sequences. Alignment bitscores were length-normalized, and a histogram plotted to investigate the distribution of the resulting 18.9 million pairwise alignments. Most of these achieve negative length-normalized scores (Fig. S1A), and by manually examining alignments at the top of the distribution we identified the 0.5 half-bit per column threshold (roughly 20-30% identity, Fig. S1B) as separating random alignments from the rare high-quality alignments between sequences too short to achieve significant E-values.

To select a coverage threshold for pham assessment, tail assembly chaperones and intein-containing genes in the training set were closely examined. Tail assembly chaperones are found in all long-tailed dsDNA phages, typically as two proteins (gpG and gpGT) produced from a single locus by a -1 or -2 ribosomal frameshift (Xu et al. 2004), such that their N-termini are identical up to the frameshift. The two proteins serve related but distinct roles in promoting proper tail tube formation so ideally, they would be placed in separate phams. For the 1,164 training genomes with two overlapping tail assembly chaperones annotated, we plotted the gpG-to-gpGT length ratio as a cumulative distribution function (Fig. S1C) and determined that a 60% coverage threshold should separate most gpG/gpGT pairs.

Inteins are selfish mobile elements found within protein-coding genes, and which splice themselves out of the translated peptide leaving the original host protein intact (Raghavan and Minnick 2009). Unlike tail assembly chaperones, intein-containing genes are relatively rare in phages (only 201 of the 89,208 non-redundant training genes have Conserved Domain Database hits to intein domains) but are typically found in well-conserved genes with intein-free homologs in related phages. Because inteins excise themselves from the translated peptide leaving the original protein intact and presumably functionally unimpaired, intein-free and intein-containing homologs would ideally be placed in the same pham. Analysis of intein size relative to the length of their host gene reveals that in phages they typically make up less than 40% of the overall length of the gene (Fig. S1D), such that a 60% coverage threshold should allow assembly of phams whose members have differential intein presence.

FIGURE LEGENDS

Figure S1. **Factors used to choose false positive similarity and coverage thresholds for parameter training**. (A) Genes from the training set with no all-versus-all BLASTP hits were globally aligned to each other (N=18.9 million alignments), and their length-normalized alignment bitscores plotted as a histogram. (B) Pairs of genes indicated by BLASTP as potential global homologs (60% bi-directional coverage, E-value less than or equal to 0.001) were globally aligned (N=1.1 million alignments) and their length-normalized alignment bitscores plotted against their percent identity to analyze the relationship between identity and alignment score. (C) The relationship between short (gpG) and long (gpGT) isoforms of tail assembly chaperones (N=1,164 chaperone pairs) was examined by dividing the length of each gpG by the length of its paired gpGT and plotting the results as a cumulative distribution function. (D) The relationship between inteins and their host proteins (N=201 intein-containing genes) was examined by dividing the size of each putative intein by the overall length of the protein it is found in, also plotted as a cumulative distribution function.

REFERENCES

Camacho, C., G. Coulouris, V. Avagyan, N. Ma, J. Papadopoulos *et al.*, 2009 BLAST+: architecture and applications. *BMC Bioinformatics* 10:421.

Marchler-Bauer, A., M.K. Derbyshire, N.R. Gonzales, S. Lu, F. Chitsaz *et al.*, 2015 CDD: NCBI's conserved domain database. *Nucleic Acids Res* 43 (Database issue):D222-226.

Raghavan, R., and M.F. Minnick, 2009 Group I introns and inteins: disparate origins but convergent parasitic strategies. *J Bacteriol* 191 (20):6193-6202.

Xu, J., R.W. Hendrix, and R.L. Duda, 2004 Conserved translational frameshift in dsDNA bacteriophage tail assembly genes. *Mol Cell* 16 (1):11-21.

Table S1. Metadata for the Actinobacteriophage genomes used to optimize MMseqs2 parameters.

PhageID[1]	Accession[2]	Host Genus	Cluster[3]	Length (bp)	# Genes	GC (%)
phiAsp2	NC_005885	Actinoplanes	Singleton	58638	76	70.4
Bennie	KU160640	Arthrobacter	AK	43075	62	61.4
Canowicakte	MF140400	Arthrobacter	AK	43914	62	61.2
Christian	MF140404	Arthrobacter	AK	43081	61	60.7
Daiboju	MH450117	Arthrobacter	AK	43950	60	61.9
Dino	MF140407	Arthrobacter	AK	43562	62	61.1
DrRobert	KU160643	Arthrobacter	AK	42601	59	60.6
Fluke	MG198781	Arthrobacter	AK	43812	62	60.9
Glenn	KU160645	Arthrobacter	AK	44389	64	60.8
Greenhouse	KX688103	Arthrobacter	AK	43977	61	60.8
Herb	MH450118	Arthrobacter	AK	43949	60	61.9
HunterDalle	KU160648	Arthrobacter	AK	43336	60	61.6
Huntingdon	MG210949	Arthrobacter	AK	43891	61	60.7
Immaculata	KU160649	Arthrobacter	AK	43661	62	61.0
Joann	KU160652	Arthrobacter	AK	44183	63	60.7
KingBob	MH450121	Arthrobacter	AK	43950	60	61.9
Korra	KU160653	Arthrobacter	AK	43707	59	61.1
Lucy	KX576641	Arthrobacter	AK	42944	60	60.7
MeganNoll	MG198782	Arthrobacter	AK	44258	63	60.9
Moki	MH744421	Arthrobacter	AK	43161	62	61.3
Nubia	MF140424	Arthrobacter	AK	44045	61	60.7
Oxynfrius	KX688102	Arthrobacter	AK	44163	62	60.8
PitaDog	MF140425	Arthrobacter	AK	42963	61	60.7
Preamble	KU160659	Arthrobacter	AK	43374	64	60.7
Pumancara	KU160661	Arthrobacter	AK	42830	61	61.7
RAP15	KU160662	Arthrobacter	AK	44259	63	60.9
RcigaStruga	KX576640	Arthrobacter	AK	43891	62	60.7
Sergei	MH450131	Arthrobacter	AK	43950	60	61.9
Suppi	KX621004	Arthrobacter	AK	43914	62	61.2
Temper16	MF668285	Arthrobacter	AK	43950	60	61.9
Urla	MG198779	Arthrobacter	AK	43940	62	61.2
Vallejo	KX621005	Arthrobacter	AK	43607	62	61.0
Vulture	KU160671	Arthrobacter	AK	43336	60	61.6
Wayne	KU160672	Arthrobacter	AK	44371	63	61.1
Laroye	KU160654	Arthrobacter	AL	60005	99	64.8
LiSara	MF140418	Arthrobacter	AL	60137	96	64.7
Salgado	KU160664	Arthrobacter	AL	59807	99	64.6
Shrooms	MF140427	Arthrobacter	AL	59707	98	64.5
Waltz	MG099950	Arthrobacter	AL	58499	96	64.3
Wheelbite	MF140434	Arthrobacter	AL	59879	94	64.8
Arcadia	MF189170	Arthrobacter	AM	58059	97	45.2
Cheesy	MF324906	Arthrobacter	AM	58739	101	45.2
Circum	KU160642	Arthrobacter	AM	58353	99	45.2
Correa	MF189171	Arthrobacter	AM	57401	96	45.2
Elsa	MF189172	Arthrobacter	AM	58059	97	45.2

Heisenberger	MF189173	Arthrobacter	AM	58208	100	45.1
KeaneyLin	MH450120	Arthrobacter	AM	57847	95	45.4
Mudcat	KU647628	Arthrobacter	AM	59443	95	45.1
Nason	MF189174	Arthrobacter	AM	58059	97	45.1
Tribby	MF189175	Arthrobacter	AM	59084	102	45.2
Azathoth	MH576948	Arthrobacter	AN	15556	26	60.1
CGermain	MH576950	Arthrobacter	AN	15556	26	60.1
Chestnut	KY434670	Arthrobacter	AN	15556	26	60.1
Copper	MH576951	Arthrobacter	AN	15556	26	60.1
Courtney3	KX443695	Arthrobacter	AN	15556	26	60.1
Decurro	KT355471	Arthrobacter	AN	15524	26	60.2
Dewayne	MH576952	Arthrobacter	AN	15556	26	60.1
Elkhorn	MF140409	Arthrobacter	AN	15556	26	59.6
Guntur	MH779506	Arthrobacter	AN	15556	26	60.1
Hunnie	MH576955	Arthrobacter	AN	15556	26	60.1
Inspire2	MH576957	Arthrobacter	AN	15556	26	60.1
Jessica	KT355473	Arthrobacter	AN	15556	26	60.1
KylieMac	MF140415	Arthrobacter	AN	15540	27	59.8
Link	MF140417	Arthrobacter	AN	15521	26	60.2
Lore	MF140419	Arthrobacter	AN	15556	26	59.6
Maggie	KU160655	Arthrobacter	AN	15556	26	60.1
Mariposa	MF140420	Arthrobacter	AN	15556	26	60.1
Massimo	KX576642	Arthrobacter	AN	15556	26	60.1
Moloch	KU160657	Arthrobacter	AN	15630	26	60.0
Muttlie	KU160658	Arthrobacter	AN	15524	26	60.2
Prospero	KX610765	Arthrobacter	AN	15556	26	60.1
Ronnie	MH576961	Arthrobacter	AN	15556	26	60.1
Sandman	KT355475	Arthrobacter	AN	15630	26	60.0
Seume	MF140426	Arthrobacter	AN	15319	26	60.3
Stratus	KU160667	Arthrobacter	AN	15630	26	60.0
Swenson	MF140429	Arthrobacter	AN	15680	26	59.9
Taj14	MF140431	Arthrobacter	AN	15546	26	59.9
TinoCrisci	MF140433	Arthrobacter	AN	15556	26	60.1
Toulouse	KU160670	Arthrobacter	AN	15319	25	60.3
TymAbreu	KT783672	Arthrobacter	AN	15556	26	60.1
Yank	KU160674	Arthrobacter	AN	15524	26	60.2
Beans	MF324907	Arthrobacter	AO1	49797	73	63.6
Brent	KT365401	Arthrobacter	AO1	49879	74	63.4
Franzy	MF377442	Arthrobacter	AO1	49976	72	63.3
Jawnski	KU160651	Arthrobacter	AO1	49419	73	63.4
Piccoletto	MF189177	Arthrobacter	AO1	49808	74	63.6
BarretLemon	KU647629	Arthrobacter	AO2	51290	79	60.9
Jordan	MF189176	Arthrobacter	AO2	51347	78	61.1
LeeroyJ	MH825703	Arthrobacter	AO2	51025	79	60.9
Martha	KU160656	Arthrobacter	AO2	51027	77	61.0
Shade	MF189178	Arthrobacter	AO2	50930	77	61.1
Sonny	KU160665	Arthrobacter	AO2	50909	77	61.1
TaeYoung	KU160668	Arthrobacter	AO2	50999	78	61.0
Timinator	MF377441	Arthrobacter	AO2	51138	79	60.9

Tank	KU160669	Arthrobacter	AP	67592	105	62.9
Wilde	KU160673	Arthrobacter	AP	68203	109	62.9
Amigo	KU160638	Arthrobacter	AQ	59173	86	52.9
Anansi	KU160639	Arthrobacter	AQ	58848	86	53.0
Gorgeous	KU160647	Arthrobacter	AQ	58979	86	53.0
Molivia	MF185731	Arthrobacter	AQ	58247	92	53.5
Rings	KU160663	Arthrobacter	AQ	59167	87	53.0
SorJuana	KU160666	Arthrobacter	AQ	58979	86	53.0
ArV1	NC_026606	Arthrobacter	AR	71200	99	61.6
Chocolat	KX670787	Arthrobacter	AR	69798	111	61.7
Chubster	KX670786	Arthrobacter	AR	70258	112	61.7
Colucci	MF185718	Arthrobacter	AR	70707	114	61.7
Conboy	KX522650	Arthrobacter	AR	70096	111	61.7
EdgarPoe	KX855961	Arthrobacter	AR	70176	111	61.6
HumptyDumpty	KX855962	Arthrobacter	AR	69978	111	61.7
JayCookie	MF668274	Arthrobacter	AR	70362	112	61.7
Kabreeze	MF185721	Arthrobacter	AR	70035	111	61.7
KBurrousTX	MH744419	Arthrobacter	AR	71020	107	62.3
PrincessTrina	KU160660	Arthrobacter	AR	70265	112	61.6
RosiePosie	MF185723	Arthrobacter	AR	70396	112	61.7
Scavito	MF185724	Arthrobacter	AR	70123	112	61.6
Tophat	MF185725	Arthrobacter	AR	70091	111	61.6
Abidatro	MF140397	Arthrobacter	AS1	39122	66	68.5
Galaxy	KU160644	Arthrobacter	AS1	37809	65	68.4
Coral	MH834606	Arthrobacter	AS2	38317	72	66.7
Cote	MH834608	Arthrobacter	AS2	39044	75	66.7
Daob	MH834609	Arthrobacter	AS2	37967	71	66.6
Kepler	MH834616	Arthrobacter	AS2	38449	75	66.7
Lunar	MH834618	Arthrobacter	AS2	38450	73	66.7
Melons	MH834620	Arthrobacter	AS2	38457	73	66.8
Polka	MH834624	Arthrobacter	AS2	38077	71	66.7
Andrew	MH834595	Arthrobacter	AS3	38802	71	65.5
KellEzio	KU647626	Arthrobacter	AT	58871	98	63.3
Kitkat	KU647627	Arthrobacter	AT	58560	100	63.4
Breylor17	MH450115	Arthrobacter	AU1	57822	84	49.9
CapnMurica	KU160641	Arthrobacter	AU1	58159	88	49.6
Caterpillar	MF140401	Arthrobacter	AU1	56622	86	51.1
ElephantMan	MF038791	Arthrobacter	AU1	58405	93	49.9
Gordon	KU160646	Arthrobacter	AU1	58279	89	49.8
MediumFry	MH450125	Arthrobacter	AU1	56978	85	50.9
Nightmare	MF140423	Arthrobacter	AU1	58839	92	49.9
Niktson	MF038790	Arthrobacter	AU1	58405	93	49.9
Synepsis	MH479926	Arthrobacter	AU1	57542	84	49.9
Tatanka	MH399789	Arthrobacter	AU1	58120	85	50.0
Teacup	MF140432	Arthrobacter	AU1	58238	88	49.8
Tenno	MH825711	Arthrobacter	AU1	58193	92	49.6
Adat	MF668266	Arthrobacter	AV	45428	56	45.7
Brad	MH450114	Arthrobacter	AV	45418	56	45.7
GurgleFerb	MF668273	Arthrobacter	AV	45426	56	45.7

Jasmine	KU160650	Arthrobacter	AV	46723	57	45.9
Nellie	MF668279	Arthrobacter	AV	45428	57	45.8
Adaia	MH834594	Arthrobacter	AX	15840	28	56.1
Atraxa	MH834597	Arthrobacter	AX	14927	23	58.0
Sputnik	MH834628	Arthrobacter	AX	14927	23	58.0
Auxilium	MH834598	Arthrobacter	AY	49447	92	62.6
Faja	MH834612	Arthrobacter	AY	52490	95	63.1
Richie	MH834625	Arthrobacter	AY	53867	98	62.7
DrManhattan	MH834610	Arthrobacter	AZ	42577	72	66.0
Liebe	MK061413	Arthrobacter	AZ	45803	69	68.8
Maureen	MH834619	Arthrobacter	AZ	45802	69	68.8
Yang	MH834629	Arthrobacter	AZ	43206	68	68.4
Bridgette	MH834603	Arthrobacter	FA	43113	71	65.1
Constance	MH834605	Arthrobacter	FA	43706	70	65.1
Eileen	MH834611	Arthrobacter	FA	41165	63	65.1
Judy	MH834614	Arthrobacter	FA	43440	72	65.2
Peas	MH834623	Arthrobacter	FA	44103	68	64.7
Corgi	MH834607	Arthrobacter	FE	15771	26	67.6
Noely	MH834622	Arthrobacter	FE	15013	23	68.3
Nandita	MH834621	Arthrobacter	FF	42378	66	64.9
Ryan	MH834627	Arthrobacter	FF	43182	70	65.1
ArV2	NC_022972	Arthrobacter	Singleton	37372	68	62.7
MargaretKali	MH450123	Arthrobacter	Singleton	39448	72	61.1
LuckyBarnes	MF668275	Brevibacterium	Singleton	50774	67	61.9
CMP1	NC_013698	Clavibacter	Singleton	58652	74	57.0
CN1A	NC_023549	Clavibacter	Singleton	56789	78	62.1
C3PO	MG198776	Corynebacterium	EN	67383	93	52.3
Darwin	MG198777	Corynebacterium	EN	67743	94	52.1
PotatoChip	MG198778	Corynebacterium	EN	66359	92	52.3
Zion	MG198780	Corynebacterium	EN	66392	92	52.3
Poushou	MF197383	Corynebacterium	EO	40353	54	60.0
TouchMeNot	MH271317	Corynebacterium	EO	40526	54	60.0
SamW	MH727560	Corynebacterium	EP	44609	60	68.1
BFK20	AJ278322	Corynebacterium	Singleton	42972	54	56.2
Juicebox	MH727550	Corynebacterium	Singleton	41394	59	67.9
P1201	NC_009816	Corynebacterium	Singleton	70579	97	50.9
JSwag	KX557280	Gordonia	A15	52726	101	61.9
KatherineG	KU998251	Gordonia	A15	52689	98	61.9
LastResort	MH513974	Gordonia	A15	52619	97	62.0
Nedarya	MH316566	Gordonia	A15	53140	97	62.0
Remus	KX557283	Gordonia	A15	52738	98	62.0
Rosalind	KU998250	Gordonia	A15	52684	99	61.9
ShayRa	MF668282	Gordonia	A15	51238	97	62.1
Soups	KU998249	Gordonia	A15	52924	99	61.9
Strosahl	KX557284	Gordonia	A15	52738	98	62.0
Waits	MH001454	Gordonia	A15	52856	98	62.0
Gustav	MG198784	Gordonia	CD	44408	69	67.9
Mahdia	MG198783	Gordonia	CD	42475	61	67.2
Morrissey	MH271305	Gordonia	CD	44924	66	65.8

Trine	MH271318	Gordonia	CD	43758	66	67.8
Bachita	KU998247	Gordonia	CQ1	93843	182	61.9
ClubL	KU998246	Gordonia	CQ1	92618	179	61.9
Cucurbita	KX557276	Gordonia	CQ1	93686	178	62.0
Lozinak	MF919520	Gordonia	CQ1	93201	179	61.9
Smoothie	KU998244	Gordonia	CQ1	93139	179	61.9
Toniann	MF919537	Gordonia	CQ1	92546	179	61.9
OneUp	KU998245	Gordonia	CQ2	93577	163	61.5
Flapper	MG757157	Gordonia	CR1	67527	96	65.3
GRU1	JF923797	Gordonia	CR1	65766	94	65.5
GTE5	JF923796	Gordonia	CR1	65839	92	65.1
Turuncu	MH744424	Gordonia	CR1	66808	94	65.4
Buggaboo	MH779499	Gordonia	CR2	68626	94	65.6
Emianna	MH779501	Gordonia	CR2	68190	95	65.8
Foxboro	MH727547	Gordonia	CR2	67773	92	65.8
GTE8	KR053201	Gordonia	CR2	67617	94	66.0
Jifall16	MH779508	Gordonia	CR2	67470	93	65.8
Kabluna	MF919510	Gordonia	CR2	65869	95	65.6
KidneyBean	MH727552	Gordonia	CR2	67802	92	65.8
Kurt	MH779510	Gordonia	CR2	68205	95	65.8
NatB6	MH536824	Gordonia	CR2	67081	93	65.7
SuperSulley	MG757166	Gordonia	CR2	64124	86	65.4
Patio	MF919542	Gordonia	CR3	66251	90	65.6
Skysand	MH669013	Gordonia	CR3	67359	95	65.5
Marietta	MH669007	Gordonia	CR4	64370	91	66.1
Fury	MH536819	Gordonia	CR5	64104	96	65.2
Pleakley	MH576960	Gordonia	CR5	64105	96	65.2
GMA7	KR063278	Gordonia	CS1	73419	101	56.6
GTE7	JN035618	Gordonia	CS1	74431	103	56.8
Adgers	MG757152	Gordonia	CS2	76008	108	59.0
BirksAndSocks	MG099940	Gordonia	CS2	77354	110	58.9
Boneham	MG757155	Gordonia	CS2	77497	109	58.9
Flakey	MG770211	Gordonia	CS2	76889	107	58.9
Hotorobo	KU963245	Gordonia	CS2	76972	108	58.9
Monty	KU998241	Gordonia	CS2	75680	105	58.9
SteveFrench	MG770214	Gordonia	CS2	75687	103	59.2
Anamika	MG099935	Gordonia	CS3	73851	92	59.2
Hail2Pitt	MH025889	Gordonia	CS3	73659	91	59.1
Woes	KU998240	Gordonia	CS3	73752	91	59.1
Benczkowski14	KU963262	Gordonia	CS4	75380	99	59.5
Demosthenes	KU998242	Gordonia	CS4	74073	95	59.3
Katyusha	KU963258	Gordonia	CS4	75380	99	59.5
Kvothe	KU998243	Gordonia	CS4	75462	99	59.5
Teatealatte	MH576976	Gordonia	CS4	75345	100	59.6
Cozz	KU998239	Gordonia	CT	46600	68	60.0
Emalyn	KU963260	Gordonia	CT	43982	67	61.2
GTE2	NC_015720	Gordonia	CT	45530	57	60.3
Margaret	MH271302	Gordonia	CT	46950	75	62.8
SketchMex	MH450132	Gordonia	CT	45847	67	60.5

Troje	MG770215	Gordonia	CT	45909	71	60.4
DinoDaryn	KY471269	Gordonia	CU1	44936	82	66.2
Huffy	KY471268	Gordonia	CU1	44936	82	66.2
Splinter	KU998238	Gordonia	CU1	45858	79	66.1
Vendetta	KU998237	Gordonia	CU1	45858	79	66.1
Gsput1	KP790011	Gordonia	CU2	43505	71	62.8
Schmidt	MH651189	Gordonia	CU4	43099	76	65.7
Blueberry	KU998236	Gordonia	CV	54990	86	67.0
CaptainKirk2	KX557274	Gordonia	CV	47898	79	67.4
CarolAnn	KX557275	Gordonia	CV	54167	80	66.9
Fenry	MH020241	Gordonia	CV	49965	78	67.2
Frokostdame	MH536818	Gordonia	CV	52531	84	66.9
Guacamole	KU963259	Gordonia	CV	49894	78	67.2
Lysidious	MF919521	Gordonia	CV	50948	83	67.0
Obliviate	KU963254	Gordonia	CV	49286	80	67.5
Petra	MH153808	Gordonia	CV	52304	89	67.1
UmaThurman	KU963251	Gordonia	CV	50127	83	67.0
Utz	KU998248	Gordonia	CV	49768	71	67.7
Zarbodnamra	MH576969	Gordonia	CV	49729	84	67.3
Jeanie	KU998256	Gordonia	CW1	17118	27	68.6
McGonagall	KU998255	Gordonia	CW1	17119	27	68.6
GMA5	KR053198	Gordonia	CW2	17562	28	66.4
GRU3	KR053197	Gordonia	CW2	17727	26	66.5
Kampe	KU998254	Gordonia	CX	80649	115	47.0
Orchid	KU998253	Gordonia	CX	80650	114	47.0
PatrickStar	KU998252	Gordonia	CX	80729	115	47.0
RobinSparkles	MH479923	Gordonia	CX	81834	124	46.9
Angelique	MH651167	Gordonia	CY1	50970	76	66.6
Confidence	MG872834	Gordonia	CY1	50646	79	66.4
Pollux	MH513979	Gordonia	CY1	52488	82	66.8
BritBrat	KU998233	Gordonia	CY2	55524	98	65.0
BatStarr	KX557273	Gordonia	CZ1	53432	83	66.6
Eviarto	MH479912	Gordonia	CZ1	53407	84	66.5
Kita	KU963257	Gordonia	CZ1	50346	80	66.7
Nymphadora	KU963255	Gordonia	CZ1	53431	84	66.6
TimTam	MH479927	Gordonia	CZ1	53431	84	66.6
Zirinka	KX557287	Gordonia	CZ1	52077	79	66.7
Attis	KU963247	Gordonia	CZ2	47881	74	66.8
Bjanes7	MG099941	Gordonia	CZ2	46042	72	66.6
Ebert	MH271295	Gordonia	CZ2	46653	78	66.6
SoilAssassin	KU963246	Gordonia	CZ2	47880	74	66.8
BaxterFox	KU963263	Gordonia	CZ3	53717	86	66.5
Yeezy	KU963249	Gordonia	CZ3	51884	86	66.7
Beenie	MG845393	Gordonia	CZ4	47953	73	66.3
Easley	MH155867	Gordonia	CZ4	46717	77	66.6
Howe	KU252585	Gordonia	CZ4	53182	80	65.6
Bowser	KU998235	Gordonia	DB	46570	67	67.1
GEazy	MH479915	Gordonia	DB	46728	73	66.9
Hedwig	KX557279	Gordonia	DB	44536	70	67.2

Schwabeltier	KU963252	Gordonia	DB	46895	71	67.0
Danyall	MH479910	Gordonia	DC	58695	95	67.8
KimmyK	MH479917	Gordonia	DC	58755	92	67.8
TillyBobJoe	MH669015	Gordonia	DC	58677	93	67.8
Twister6	KX557286	Gordonia	DC	57804	93	67.7
Wizard	KU998234	Gordonia	DC	58308	89	67.9
Angelicage	MH651166	Gordonia	DE1	59215	84	67.4
Ashertheman	MH651168	Gordonia	DE1	57555	85	68.0
Brandonk123	MG812487	Gordonia	DE1	58842	89	67.3
Lennon	MF919514	Gordonia	DE1	60237	85	67.4
Ribeye	MH450129	Gordonia	DE1	57448	85	68.1
Rofo	MH479924	Gordonia	DE1	58767	85	67.3
Sitar	MH153809	Gordonia	DE1	59641	84	67.3
Vivi2	KU963250	Gordonia	DE1	59337	89	67.1
Ali17	MH669000	Gordonia	DE2	58301	84	68.4
Phinally	KU963253	Gordonia	DE2	59265	87	68.4
GTE6	KR053200	Gordonia	DE3	56982	86	67.8
Gmala1	KP790009	Gordonia	DF1	75167	90	50.8
GordDuk1	KP790010	Gordonia	DF1	76276	97	50.7
GordTnk2	KP790008	Gordonia	DF1	75987	98	50.7
GMA3	KR063279	Gordonia	DF2	77779	104	51.3
Jumbo	KX557281	Gordonia	DF3	78302	102	54.5
Beyoncage	MH576972	Gordonia	DG	66610	96	59.6
Djokovic	MH025890	Gordonia	DG	66609	96	59.6
Suzy	MH271313	Gordonia	DG	67118	96	59.1
Terapin	KX557285	Gordonia	DG	66611	97	59.6
Lucky10	KU963256	Gordonia	DH	42979	70	65.4
BetterKatz	KU963261	Gordonia	DI	50636	75	67.1
DelRio	MH509446	Gordonia	DI	50961	75	67.0
Nadeem	MH399781	Gordonia	DI	49897	74	67.3
Gravy	MG962368	Gordonia	DJ	59545	93	51.1
Kerry	MG962369	Gordonia	DJ	59608	93	51.1
Bantam	KX557272	Gordonia	DL	92580	168	64.7
Daredevil	MH590603	Gordonia	DL	90490	165	64.8
Emperor	MH271296	Gordonia	DM	16604	24	70.1
SallySpecial	MG812496	Gordonia	DM	15896	21	70.1
BENtherdunthat	MG099939	Gordonia	DN1	54867	102	63.4
Getalong	MH779504	Gordonia	DN1	56214	104	62.8
Horus	MH651176	Gordonia	DN1	55668	103	63.0
Phistory	MH651185	Gordonia	DN1	53085	106	63.0
Apricot	MH536812	Gordonia	DN3	52195	101	63.1
Fryberger	MH479913	Gordonia	DP	67234	144	50.2
Ronaldo	MH479925	Gordonia	DP	68389	147	50.2
GMA6	KR063280	Gordonia	DQ	83324	115	58.2
Sour	MH153810	Gordonia	DR	61670	79	68.0
GMA2	KR063281	Gordonia	DS	103424	126	53.4
Nyceirae	KX557282	Gordonia	DT	41857	60	67.5
Neville	MH651182	Gordonia	DU	75813	129	58.8
Catfish	MH697580	Gordonia	Singleton	46888	78	65.0

Eyre	KX557277	Gordonia	Singleton	44929	74	67.5
GAL1	KR053194	Gordonia	Singleton	49979	82	63.5
Ghobes	KX557278	Gordonia	Singleton	45285	59	65.2
GMA1	KR053195	Gordonia	Singleton	41207	68	65.7
GMA4	KR053199	Gordonia	Singleton	45537	68	66.4
Ruthy	MH536826	Gordonia	Singleton	51265	73	66.7
Yvonnetastic	KU963248	Gordonia	Singleton	98136	198	59.7
AlexAdler	MG962360	Microbacterium	EA1	41834	63	63.4
Antoinette	MH045565	Microbacterium	EA1	41858	63	63.4
Aubergine	MG839015	Microbacterium	EA1	41555	62	63.4
AxiPup	MG839016	Microbacterium	EA1	41770	63	63.3
Baines	MG839017	Microbacterium	EA1	41555	62	63.4
Balsa	MG839030	Microbacterium	EA1	41862	62	63.4
Bandik	MH045554	Microbacterium	EA1	41804	63	63.5
BeeBee8	MH045555	Microbacterium	EA1	42027	65	63.3
Bonino	MG920061	Microbacterium	EA1	41534	62	63.4
Dave	MH045558	Microbacterium	EA1	41858	63	63.4
Espinosa	MG839018	Microbacterium	EA1	41553	62	63.4
Etna	MH045559	Microbacterium	EA1	41908	64	63.4
Gargoyle	MH153802	Microbacterium	EA1	41803	63	63.5
Gelo	MG962367	Microbacterium	EA1	41562	62	63.4
Hamlet	MG839019	Microbacterium	EA1	41934	63	63.4
Ilzat	MG839029	Microbacterium	EA1	41525	62	63.5
Kale	MG839020	Microbacterium	EA1	41558	62	63.4
Knox	MG839021	Microbacterium	EA1	41797	63	63.3
Ludgate	MG839022	Microbacterium	EA1	41799	63	63.5
Martin	MH153805	Microbacterium	EA1	41812	63	63.5
Nagem	MH045560	Microbacterium	EA1	41846	63	63.5
Nattles	MG925352	Microbacterium	EA1	41544	62	63.4
Oats	MH153806	Microbacterium	EA1	41555	62	63.4
Papafritta	MH513981	Microbacterium	EA1	41573	62	63.4
Peep	MG839023	Microbacterium	EA1	41856	63	63.4
Peppino	MG839024	Microbacterium	EA1	41932	63	63.3
PuppyEggo	MG944219	Microbacterium	EA1	41803	63	63.3
Raccoon	MG839025	Microbacterium	EA1	41894	63	63.4
Raptor	MH045562	Microbacterium	EA1	41801	63	63.4
Redfield	MH479922	Microbacterium	EA1	41930	63	63.3
Robinson	MH045563	Microbacterium	EA1	41874	63	63.5
Schnapsidee	MH590590	Microbacterium	EA1	41872	63	63.4
StingRay	MG944222	Microbacterium	EA1	41597	62	63.5
Superfresh	MG839026	Microbacterium	EA1	41860	63	63.4
Teagan	MH153811	Microbacterium	EA1	41793	63	63.5
TeddyBear	MH045564	Microbacterium	EA1	41555	62	63.4
Tenda	MG839028	Microbacterium	EA1	41553	62	63.4
Andromedas	MH590606	Microbacterium	EA2	40494	63	62.0
ColaCorta	MH590604	Microbacterium	EA2	40494	63	62.0
Eleri	MG839027	Microbacterium	EA2	40366	63	62.0
Sansa	MH513982	Microbacterium	EA2	40306	61	61.8
Casey	MG944226	Microbacterium	EA3	39307	59	61.3

Pajaza	MG944216	Microbacterium	EA3	39307	59	61.2
Pikmin	MG944218	Microbacterium	EA3	39307	59	61.3
Golden	MG925343	Microbacterium	EA4	39640	58	64.1
Koji	MG925345	Microbacterium	EA4	39403	56	64.2
Lucky3	MG925347	Microbacterium	EA4	39640	58	64.1
Neferthena	MH697589	Microbacterium	EA5	41706	62	64.4
Armstrong	MH834596	Microbacterium	EB	39928	68	67.1
Bernstein	MH834599	Microbacterium	EB	39926	68	67.1
Brahms	MH834602	Microbacterium	EB	39828	68	67.1
Coltrane	MH834604	Microbacterium	EB	39828	68	67.1
Didgeridoo	MH045566	Microbacterium	EB	42655	75	66.1
Dismas	MG670586	Microbacterium	EB	41593	66	69.6
Eden	MH509447	Microbacterium	EB	40833	69	66.3
Elva	MH045567	Microbacterium	EB	42139	71	68.2
Kieran	MH045568	Microbacterium	EB	41417	63	69.7
Rollins	MH834626	Microbacterium	EB	39926	68	67.1
KaiHaiDragon	MH590600	Microbacterium	EC	52992	91	68.9
Metamorphoo	MH271304	Microbacterium	EC	54148	92	68.5
Paschalis	MH155873	Microbacterium	EC	52935	90	68.8
Quhwah	MH271321	Microbacterium	EC	53549	94	68.8
RobsFeet	MH271312	Microbacterium	EC	54189	97	68.6
Hortus1	MH271300	Microbacterium	ED1	63119	114	64.6
Jacko	MH399779	Microbacterium	ED1	61421	115	65.0
OlinDD	MH271307	Microbacterium	ED1	63123	114	64.6
Pioneer3	MH271310	Microbacterium	ED1	62954	114	64.6
Tandem	MH271316	Microbacterium	ED1	63128	114	64.7
Fork	MH371108	Microbacterium	ED2	62090	117	61.8
Lyell	MH371109	Microbacterium	ED2	62716	122	61.6
Musetta	MH536823	Microbacterium	ED2	63604	119	61.7
BonaeVitae	MH045556	Microbacterium	EE	17451	27	68.2
BurtonThePup	MH045557	Microbacterium	EE	17445	25	68.8
Dongwon	MH744416	Microbacterium	EE	17362	25	68.5
Efeko	MH825700	Microbacterium	EE	17491	28	68.6
KayPaulus	MH371118	Microbacterium	EE	17455	25	68.5
Miaurora	MH779512	Microbacterium	EE	17032	25	69.0
Minima	MH651181	Microbacterium	EE	17362	25	68.5
Noelani	MH399783	Microbacterium	EE	17349	25	68.2
PaoPu	MH045561	Microbacterium	EE	17362	25	68.5
Quaker	MH371111	Microbacterium	EE	17450	25	68.6
Scamander	MH576963	Microbacterium	EE	17452	25	68.7
VitulaEligans	MH371124	Microbacterium	EE	17534	25	68.8
AnnaSerena	MH271292	Microbacterium	EF	56707	83	63.8
Krampus	MH271301	Microbacterium	EF	56708	83	63.8
Hyperion	MH153803	Microbacterium	EG	61769	104	67.0
OneinaGillian	MH727556	Microbacterium	EG	61703	103	67.1
Squash	MH153813	Microbacterium	EG	62312	108	66.8
Floof	MH271298	Microbacterium	EH	48500	80	69.0
Percival	MH271308	Microbacterium	EH	47364	74	69.6
MementoMori	MH271303	Microbacterium	EI	55572	96	70.0

Appa	MH153799	Microbacterium	Singleton	38684	65	68.0
Burro	MH825698	Microbacterium	Singleton	54473	49	64.3
Camille	MH153800	Microbacterium	Singleton	53097	77	56.3
Count	MH153801	Microbacterium	Singleton	78922	131	51.4
Hendrix	MH183162	Microbacterium	Singleton	97757	154	66.1
Min1	NC_009603	Microbacterium	Singleton	46365	77	68.3
Triscuit	MH047631	Microbacterium	Singleton	67539	112	58.3
ValentiniPuff	MH825712	Microbacterium	Singleton	62517	112	67.1
Zeta1847	MH271320	Microbacterium	Singleton	47921	75	71.4
ISF9	KJ173786	Microbacterium	UNK	59256	120	62.8
Abrogate	KM597531	Mycobacterium	A1	52530	91	63.8
Acme	MG812486	Mycobacterium	A1	51793	91	63.5
Adahisdi	MH697575	Mycobacterium	A1	51703	84	63.8
Aeneas	JQ809703	Mycobacterium	A1	53684	99	63.6
Alsfro	KJ174156	Mycobacterium	A1	52136	98	63.6
Alvin	KP027205	Mycobacterium	A1	49577	86	63.6
Arcanine	MH744414	Mycobacterium	A1	52273	94	63.7
Arlo	MH576971	Mycobacterium	A1	52960	96	63.8
Barriga	KR997929	Mycobacterium	A1	52643	101	63.4
BeesKnees	MG872833	Mycobacterium	A1	51702	95	63.7
Bethlehem	AY500153	Mycobacterium	A1	52250	87	63.2
Bigfoot	KX702320	Mycobacterium	A1	51646	87	63.7
BigMau	MH450113	Mycobacterium	A1	52632	91	63.7
BillKnuckles	JN699000	Mycobacterium	A1	51821	86	63.4
Bircsak	KX522649	Mycobacterium	A1	53622	91	63.6
Blue	KY224001	Mycobacterium	A1	50864	84	63.9
Bob3	MG920060	Mycobacterium	A1	52233	94	63.8
BPBiebs31	JF957057	Mycobacterium	A1	53171	96	63.4
Bruns	JN698998	Mycobacterium	A1	53003	96	63.6
Bxb1	AF271693	Mycobacterium	A1	50550	86	63.6
CactusRose	KX683876	Mycobacterium	A1	52659	97	63.8
ConceptII	MH230876	Mycobacterium	A1	53287	97	63.5
Corvo	MG925340	Mycobacterium	A1	53325	94	63.7
Crispicous1	MH697581	Mycobacterium	A1	50827	89	63.7
DD5	EU744252	Mycobacterium	A1	51621	87	63.4
Doom	JN153085	Mycobacterium	A1	51421	85	63.8
Dreamboat	JN660814	Mycobacterium	A1	51083	94	63.9
DrFeelGood	MH536816	Mycobacterium	A1	50155	85	63.9
Dynamix	KT626047	Mycobacterium	A1	50628	91	63.7
Edtherson	KP027196	Mycobacterium	A1	51492	91	64.0
Euphoria	JN153086	Mycobacterium	A1	53597	94	63.7
Fajezeel	MG872836	Mycobacterium	A1	51083	90	63.4
Fascinus	MH000606	Mycobacterium	A1	52157	86	63.7
Forsytheast	MG925342	Mycobacterium	A1	52695	96	63.7
GageAP	MH020236	Mycobacterium	A1	51904	91	63.5
Gompeii16	KX522943	Mycobacterium	A1	53623	91	63.6
Graduation	KF560331	Mycobacterium	A1	52823	97	63.5
Greg	MG812489	Mycobacterium	A1	51083	90	63.4
HanShotFirst	KF493880	Mycobacterium	A1	52390	91	63.8

Homines	MH632117	Mycobacterium	A1	48502	87	63.8
Hope4ever	MH513971	Mycobacterium	A1	50455	88	63.6
Ichabod	MG925344	Mycobacterium	A1	53317	95	63.8
ILeeKay	MF919508	Mycobacterium	A1	51017	91	64.0
Jasper	EU744251	Mycobacterium	A1	50968	94	63.7
JC27	JF937099	Mycobacterium	A1	52169	97	63.6
KBG	EU744248	Mycobacterium	A1	53572	89	63.6
KSSJEB	JF937110	Mycobacterium	A1	51381	92	63.6
Kugel	JN699016	Mycobacterium	A1	52379	95	63.8
Kykar	MG962370	Mycobacterium	A1	50294	87	63.7
Lamina13	KJ409696	Mycobacterium	A1	53255	96	63.7
Lesedi	JF937100	Mycobacterium	A1	50486	89	63.8
Lockley	EU744249	Mycobacterium	A1	51478	90	63.4
Lopton	MH020244	Mycobacterium	A1	52394	87	63.5
Magnar	MH371110	Mycobacterium	A1	51428	91	63.7
Magnito	KX550443	Mycobacterium	A1	51743	92	63.8
Makemake	KX369584	Mycobacterium	A1	52496	93	63.8
Marcell	JX307705	Mycobacterium	A1	49186	83	64.0
Maroc7	MH651180	Mycobacterium	A1	53350	92	63.5
McGuire	MG962372	Mycobacterium	A1	51521	92	63.7
Michley	MH338238	Mycobacterium	A1	51527	92	63.7
Moose	MH479919	Mycobacterium	A1	52695	95	63.7
MPlant7149	MG925351	Mycobacterium	A1	51372	92	63.7
MrGordo	JN020140	Mycobacterium	A1	50988	92	63.8
Mryolo	MH338239	Mycobacterium	A1	50505	87	64.0
Museum	JF937103	Mycobacterium	A1	51426	90	63.6
NEHalo	MH590595	Mycobacterium	A1	50535	88	63.8
Nerujay	KR080201	Mycobacterium	A1	53455	97	63.7
Nhonho	KR997934	Mycobacterium	A1	51355	88	63.8
Niza	MH399782	Mycobacterium	A1	51672	89	63.4
Oogway	MH230878	Mycobacterium	A1	51745	87	63.6
PacerPaul	KX574454	Mycobacterium	A1	52732	93	63.6
Papez	KX369586	Mycobacterium	A1	52501	94	63.8
Pari	KT438500	Mycobacterium	A1	50614	91	63.5
PattyP	KC661273	Mycobacterium	A1	52057	92	63.6
Pepe	KT818595	Mycobacterium	A1	50515	86	64.0
Perseus	JN572689	Mycobacterium	A1	53142	92	63.7
Petruchio	KY213952	Mycobacterium	A1	49531	90	63.8
PherrisBueller	MH669011	Mycobacterium	A1	49209	88	64.0
PhrostyMug	KF279415	Mycobacterium	A1	53636	97	63.7
Pinto	KJ690250	Mycobacterium	A1	50610	87	63.5
Pippin	MG812495	Mycobacterium	A1	52034	97	63.8
Pita2	MH576959	Mycobacterium	A1	51486	91	63.4
RidgeCB	JN398369	Mycobacterium	A1	50844	94	64.0
Rohr	MH450130	Mycobacterium	A1	53483	92	63.6
Rufus	KT259047	Mycobacterium	A1	52357	93	63.9
Ruotula	MG944220	Mycobacterium	A1	52601	94	63.4
SarFire	KF024726	Mycobacterium	A1	53701	99	63.8
Scowl	MG944221	Mycobacterium	A1	51690	91	63.8

Seabiscuit	KJ194585	Mycobacterium	A1	51781	94	63.7
Sibs6	MH576974	Mycobacterium	A1	50210	95	63.8
SkiPole	GU247132	Mycobacterium	A1	53137	102	63.6
Smairt	MF668283	Mycobacterium	A1	54655	100	63.6
Smeagol	MH001458	Mycobacterium	A1	53134	90	63.7
Solon	EU826470	Mycobacterium	A1	49487	86	63.8
SwissCheese	MH450133	Mycobacterium	A1	51822	95	63.5
Switzer	JF937108	Mycobacterium	A1	52298	91	63.8
Target	MH576975	Mycobacterium	A1	49097	90	63.6
Tasp14	KT326768	Mycobacterium	A1	51409	89	63.9
TheloniousMonk	KT363731	Mycobacterium	A1	52055	90	63.6
Thor	KP027204	Mycobacterium	A1	53058	98	63.7
Treddle	KP027203	Mycobacterium	A1	53008	94	63.9
Trouble	KF024724	Mycobacterium	A1	52102	94	63.6
Turj99	KT388014	Mycobacterium	A1	51161	86	63.7
U2	AY500152	Mycobacterium	A1	51277	81	63.7
Violet	JN687951	Mycobacterium	A1	52481	90	63.8
Wheeler	KF416340	Mycobacterium	A1	53588	96	63.3
Wilkins	MG099951	Mycobacterium	A1	50907	94	63.9
Zeeculate	MH632118	Mycobacterium	A1	53828	91	63.8
Zephyr	KX712238	Mycobacterium	A1	52320	92	63.9
Goose	JX307704	Mycobacterium	A10	50645	86	65.1
KittenMittens	MH001459	Mycobacterium	A10	49894	78	64.5
OKCentral2016	MF773750	Mycobacterium	A10	50072	83	65.1
Rebeuca	JX411619	Mycobacterium	A10	51235	86	65.1
RhynO	KJ156985	Mycobacterium	A10	46739	73	65.3
Severus	KC661279	Mycobacterium	A10	49894	80	64.4
Trike	KM101120	Mycobacterium	A10	44716	65	64.5
Twister	JQ512844	Mycobacterium	A10	51094	87	65.0
Ebony	MH338236	Mycobacterium	A11	52152	98	63.8
Et2Brutus	MF140410	Mycobacterium	A11	52497	101	63.8
Jabith	KY471460	Mycobacterium	A11	52715	99	63.7
Joselito	MH338237	Mycobacterium	A11	52347	97	63.7
Mulciber	KU695581	Mycobacterium	A11	52428	98	63.7
Snape	MH536828	Mycobacterium	A11	52138	99	63.7
DarthPhader	KX657793	Mycobacterium	A12	53432	91	63.2
Steamy	MH513984	Mycobacterium	A12	53420	90	63.4
Phlei	KT206225	Mycobacterium	A13	50418	81	60.1
Luchador	KR080193	Mycobacterium	A14	53387	96	62.1
EagleEye	KF861510	Mycobacterium	A16	52974	95	61.4
40AC	KJ192196	Mycobacterium	A17	53396	90	63.3
MyraDee	MF141539	Mycobacterium	A18	50514	94	62.7
Kimona	MF472895	Mycobacterium	A19	50283	87	64.4
20ES	KJ410132	Mycobacterium	A2	53124	96	63.4
AbbyPaige	MH077576	Mycobacterium	A2	53225	96	63.4
Acolyte	MH509441	Mycobacterium	A2	52668	92	63.3
Adzzy	KF416344	Mycobacterium	A2	52519	96	62.6
AnnaL29	KF024721	Mycobacterium	A2	53253	96	64.4
Anselm	MF919490	Mycobacterium	A2	53545	98	63.5

ArcherNM	KU761559	Mycobacterium	A2	52561	91	64.2
Bactobuster	KU568494	Mycobacterium	A2	52129	87	63.1
Baehexic	KX683875	Mycobacterium	A2	53111	97	63.5
BobSwaget	MF185727	Mycobacterium	A2	50400	88	63.3
Changeling	MF140403	Mycobacterium	A2	52991	92	64.6
Che12	DQ398043	Mycobacterium	A2	52047	98	62.9
ChipMunk	KU985095	Mycobacterium	A2	53932	94	63.1
CRB1	KJ410134	Mycobacterium	A2	52963	94	63.2
Crucio	MH338234	Mycobacterium	A2	53256	95	63.5
D29	AF022214	Mycobacterium	A2	49136	77	63.5
Dalmatian	MF919500	Mycobacterium	A2	52850	95	63.5
Drake55	MF668269	Mycobacterium	A2	52719	96	63.4
DudeLittle	KX576639	Mycobacterium	A2	52917	94	63.5
Echild	KF981601	Mycobacterium	A2	53159	100	63.7
Equemioh13	KJ959632	Mycobacterium	A2	53042	96	63.5
EvilGenius	KU985093	Mycobacterium	A2	53935	93	63.0
Fameo	MH051252	Mycobacterium	A2	52618	94	63.4
First	JX899358	Mycobacterium	A2	53028	95	63.4
Flare16	MH825701	Mycobacterium	A2	52858	97	63.5
Heffalump	KY965065	Mycobacterium	A2	53085	92	63.6
Jaan	KX758539	Mycobacterium	A2	52462	90	63.2
Jerm	KX619650	Mycobacterium	A2	53163	95	63.3
JoshKayV	MF472896	Mycobacterium	A2	53366	97	63.0
Journey13	KY798216	Mycobacterium	A2	48502	81	62.7
Jsquared	MH020237	Mycobacterium	A2	52967	101	62.6
Kalpine	KX641263	Mycobacterium	A2	53330	96	64.4
Kerberos	KX758538	Mycobacterium	A2	52753	93	63.5
L5	Z18946	Mycobacterium	A2	52297	90	62.3
LadyBird	KT588442	Mycobacterium	A2	53141	96	63.5
Larenn	KM677210	Mycobacterium	A2	52967	92	63.5
LilTurb	MH825704	Mycobacterium	A2	53153	95	63.3
Lokk	MF324899	Mycobacterium	A2	51008	89	63.4
Loser	KU761558	Mycobacterium	A2	53486	97	64.5
MissWhite	MF472893	Mycobacterium	A2	50263	92	63.3
NaSiaTalie	KU297783	Mycobacterium	A2	52920	98	63.4
NicoleTera	MH825709	Mycobacterium	A2	52944	93	64.0
Odin	KF017927	Mycobacterium	A2	52807	94	62.3
Ph8s	MG099947	Mycobacterium	A2	52874	97	62.6
Piro94	KM197169	Mycobacterium	A2	52647	94	63.4
Pomar16	KX574455	Mycobacterium	A2	52833	93	63.4
Power	KM463009	Mycobacterium	A2	53395	95	63.5
Pukovnik	EU744250	Mycobacterium	A2	52892	88	63.3
QueenBeesly	MH697591	Mycobacterium	A2	53348	95	63.5
RedRock	GU339467	Mycobacterium	A2	53332	95	64.5
Serenity	KT381276	Mycobacterium	A2	52088	97	62.6
SnapTap	MF919531	Mycobacterium	A2	53051	95	63.5
StarStuff	KX897981	Mycobacterium	A2	52785	93	63.5
SweetiePie	KM591906	Mycobacterium	A2	53184	95	63.5
TipsytheTRex	MF919536	Mycobacterium	A2	52350	93	62.5

Tomathan	MH513985	Mycobacterium	A2	52876	93	63.4
Trixie	JN408461	Mycobacterium	A2	53526	92	64.5
Turbido	JN408460	Mycobacterium	A2	53169	94	63.3
Updawg	MF919538	Mycobacterium	A2	53043	97	63.5
Aglet	KX507362	Mycobacterium	A3	50866	91	64.1
Anubis	KF279418	Mycobacterium	A3	50854	93	64.0
AugsMagnumOpus	MH536813	Mycobacterium	A3	48545	87	64.2
BabyRay	KX683423	Mycobacterium	A3	50657	92	63.6
Beauxregard13	MH155864	Mycobacterium	A3	50884	89	64.0
BreSam8	MH576949	Mycobacterium	A3	50843	90	64.0
BuzzBuzz	KX523125	Mycobacterium	A3	50897	83	64.2
Bxz2	AY129332	Mycobacterium	A3	50913	86	64.2
DaHudson	KT359365	Mycobacterium	A3	50841	89	64.1
EpicPhail	MG925341	Mycobacterium	A3	50884	91	64.0
Farber	KM233455	Mycobacterium	A3	49190	84	64.2
Fred313	MF373840	Mycobacterium	A3	50053	85	64.0
Grum1	MH399776	Mycobacterium	A3	50883	90	64.1
HelDan	JF957058	Mycobacterium	A3	50364	89	64.0
Hercules11	KU985091	Mycobacterium	A3	50892	90	64.0
Hookmount	MH316563	Mycobacterium	A3	50061	88	64.1
Idleandcovert	KY464936	Mycobacterium	A3	49156	84	63.8
Isca	MN586063	Mycobacterium	A3	49968	86	64.2
JenCasNa	KU255188	Mycobacterium	A3	50877	91	64.0
JHC117	JF704098	Mycobacterium	A3	50877	85	64.0
Jobu08	KC661281	Mycobacterium	A3	50679	85	64.0
KADY	MF185729	Mycobacterium	A3	50898	87	64.2
Kalnoky	MH450119	Mycobacterium	A3	50571	89	64.0
Lambert1	MG812492	Mycobacterium	A3	50042	88	64.1
Lilith	MH020238	Mycobacterium	A3	50841	87	64.1
Louie6	KX670788	Mycobacterium	A3	50899	89	64.2
LugYA	MG757161	Mycobacterium	A3	50878	93	64.0
MadMarie	MH651179	Mycobacterium	A3	50849	87	64.0
Malinsilva	KU984914	Mycobacterium	A3	50839	93	64.0
Margo	KX640831	Mycobacterium	A3	50087	88	64.0
Marie	KU578077	Mycobacterium	A3	50877	88	64.0
Marius	MH450124	Mycobacterium	A3	50570	89	64.0
MarQuardt	KM233454	Mycobacterium	A3	50882	90	64.0
Methuselah	KC661272	Mycobacterium	A3	50891	83	64.2
Microwolf	JF704101	Mycobacterium	A3	50864	85	64.0
Misomonster	MH513975	Mycobacterium	A3	48504	86	64.3
MuchMore	MH727554	Mycobacterium	A3	48545	87	64.2
OlanP	MG925355	Mycobacterium	A3	50755	87	64.0
Ollie	MH536825	Mycobacterium	A3	50721	90	64.0
Panamaxus	MH020248	Mycobacterium	A3	50080	83	64.1
Penny1	KX458237	Mycobacterium	A3	50884	90	64.0
PGHhamlin	MH077583	Mycobacterium	A3	50840	92	64.0
Phantastic	KJ510415	Mycobacterium	A3	50101	91	63.8
PhishRPhriends	MH450126	Mycobacterium	A3	49756	85	63.9
Phoxy	KP017310	Mycobacterium	A3	49267	85	64.2

Phranny	MH371121	Mycobacterium	A3	48024	81	64.0
Pistachio	MH047633	Mycobacterium	A3	50006	88	63.8
Puppy	MH047632	Mycobacterium	A3	50084	89	63.8
PurpleHaze	KY965063	Mycobacterium	A3	48596	83	64.0
QuinnKiro	KM592966	Mycobacterium	A3	50066	87	64.0
Reba	MH450128	Mycobacterium	A3	49412	84	64.0
Rockstar	JF704111	Mycobacterium	A3	47780	78	64.3
Sabia	MH338240	Mycobacterium	A3	50855	87	64.0
Sabinator	KX808129	Mycobacterium	A3	50883	88	64.0
Spike509	KP017311	Mycobacterium	A3	50989	91	64.1
Stagni	MF185732	Mycobacterium	A3	50856	86	64.0
StepMih	MF185733	Mycobacterium	A3	50841	90	64.0
Taurus	KP027202	Mycobacterium	A3	50877	91	64.0
Texage	KT326767	Mycobacterium	A3	50081	86	64.0
Tiffany	KM101119	Mycobacterium	A3	50768	89	64.0
TNguyen7	MH051259	Mycobacterium	A3	50413	91	63.8
Todacoro	KX670828	Mycobacterium	A3	50066	88	64.0
Vix	JF704114	Mycobacterium	A3	50963	85	64.0
Watson	KX664448	Mycobacterium	A3	50841	89	64.0
Wooldri	KT381277	Mycobacterium	A3	50797	92	64.0
Abdiel	KY083058	Mycobacterium	A4	51381	88	63.9
Achebe	KY083059	Mycobacterium	A4	51433	86	63.7
Albee	KX817175	Mycobacterium	A4	51372	88	63.9
Annyong	MH051247	Mycobacterium	A4	51418	87	63.9
Arturo	JX307702	Mycobacterium	A4	51500	86	64.1
Avle17	MH509443	Mycobacterium	A4	51366	88	63.9
Backyardigan	JF704093	Mycobacterium	A4	51308	84	63.7
Badger	KX550442	Mycobacterium	A4	51274	85	63.7
BellusTerra	KF841475	Mycobacterium	A4	51236	89	63.9
Blackmoor	MG099942	Mycobacterium	A4	51374	87	63.9
Broseidon	KX808130	Mycobacterium	A4	51374	88	63.9
Bruiser	KY083060	Mycobacterium	A4	51374	88	63.9
BubbleTrouble	KY204244	Mycobacterium	A4	51397	88	63.9
Burger	KU985094	Mycobacterium	A4	51371	86	63.9
Caelakin	KT716493	Mycobacterium	A4	51374	87	63.9
Camperdownii	KY204245	Mycobacterium	A4	51131	87	63.9
Cerulean	MF919496	Mycobacterium	A4	51809	92	63.9
ChampagnePapi	MH651170	Mycobacterium	A4	51811	92	63.9
Cindaradix	MF919498	Mycobacterium	A4	51575	84	64.0
Clarenza	KY204246	Mycobacterium	A4	51372	88	63.9
Cocoaberry	KX648373	Mycobacterium	A4	51376	89	64.0
Commander	MH479909	Mycobacterium	A4	51369	86	63.9
Dhanush	KC661271	Mycobacterium	A4	51373	88	63.9
Druantia	MH536817	Mycobacterium	A4	51343	87	63.8
Eagle	HM152766	Mycobacterium	A4	51436	87	63.9
Eapen	MH479911	Mycobacterium	A4	51366	89	63.9
Eris	KY083063	Mycobacterium	A4	51386	88	64.0
Florean	KX557234	Mycobacterium	A4	51371	88	63.9
Flux	JQ809701	Mycobacterium	A4	51370	89	63.9

Funston	KY204247	Mycobacterium	A4	51372	88	64.0
Gadost	KP027198	Mycobacterium	A4	51376	88	64.0
HamSlice	KP057620	Mycobacterium	A4	51370	84	63.9
Holli	KU985092	Mycobacterium	A4	51162	88	63.9
Houdini22	MH399777	Mycobacterium	A4	51376	89	64.0
ICleared	JQ896627	Mycobacterium	A4	51440	88	63.9
Iracema64	KU055616	Mycobacterium	A4	51637	88	64.0
Jaykayelowell	MH697588	Mycobacterium	A4	51367	86	63.9
JetBlade	MF919509	Mycobacterium	A4	51374	90	63.9
JoongJeon	KY204248	Mycobacterium	A4	51366	88	63.9
Kampy	KJ510414	Mycobacterium	A4	51378	88	63.9
KFPoly	KY204249	Mycobacterium	A4	51365	88	63.9
Kratark	KY204250	Mycobacterium	A4	51407	88	63.9
LeoAvram	KY006473	Mycobacterium	A4	47079	75	63.8
LHTSCC	JN699015	Mycobacterium	A4	51813	91	63.9
LittleB	KX817174	Mycobacterium	A4	51373	88	63.9
LittleGuy	KX550441	Mycobacterium	A4	51178	88	63.9
Maverick	KT365397	Mycobacterium	A4	51372	91	63.9
Medusa	KF024733	Mycobacterium	A4	51384	87	63.9
MeeZee	JN243856	Mycobacterium	A4	51368	87	63.9
Melvin	KF841476	Mycobacterium	A4	51369	91	63.9
Millski	KY083062	Mycobacterium	A4	51374	88	63.9
Morpher26	MF668278	Mycobacterium	A4	51294	86	63.7
Morrow	MG962373	Mycobacterium	A4	51411	91	63.8
Mundrea	KX579975	Mycobacterium	A4	51257	87	64.0
Nemo27	MH077581	Mycobacterium	A4	51372	88	63.9
NorthStar	MH230877	Mycobacterium	A4	51374	86	63.9
NotAPhaseMom	MH399785	Mycobacterium	A4	51376	89	64.0
Nyxis	KF954506	Mycobacterium	A4	51250	87	63.9
Obama12	KF959563	Mycobacterium	A4	51797	89	64.0
Palestino	KY083061	Mycobacterium	A4	51369	88	63.9
Peaches	GQ303263	Mycobacterium	A4	51376	86	63.9
Phacado	MG944217	Mycobacterium	A4	51373	88	63.9
Pipcraft	MH155875	Mycobacterium	A4	51376	85	63.9
Relief	MH155879	Mycobacterium	A4	51368	87	63.9
Romney	KU867906	Mycobacterium	A4	51370	87	63.9
Roosevelt	KX619651	Mycobacterium	A4	51502	87	63.9
Ruin	MH825710	Mycobacterium	A4	51365	88	63.9
Sabertooth	JX307703	Mycobacterium	A4	51377	88	63.9
Shaka	JF792674	Mycobacterium	A4	51369	86	63.9
Skipitt	MH020249	Mycobacterium	A4	51365	87	63.9
Stasia	KX641260	Mycobacterium	A4	51591	85	63.6
Thanksgivukkah	KY204251	Mycobacterium	A4	51370	88	63.9
TinaFeyge	KY471266	Mycobacterium	A4	51367	86	63.9
Tinybot	KY549155	Mycobacterium	A4	51402	89	64.0
TiroTheta9	JN561150	Mycobacterium	A4	51367	87	63.9
Wander	MG944224	Mycobacterium	A4	51366	88	63.9
Wilbur	KX458236	Mycobacterium	A4	51370	88	63.9
Wile	JN243857	Mycobacterium	A4	51308	85	63.7

Wizard007	MH727563	Mycobacterium	A4	51029	86	63.9
AgentM	MG099934	Mycobacterium	A5	50503	85	60.9
Airmid	JN083853	Mycobacterium	A5	51241	89	60.0
Aragog	MG099937	Mycobacterium	A5	50812	86	60.8
Archetta	MG099938	Mycobacterium	A5	47409	75	60.6
Benedict	JN083852	Mycobacterium	A5	51083	90	59.8
Chadwick	KT246486	Mycobacterium	A5	49421	88	59.8
Conspiracy	KF560330	Mycobacterium	A5	50755	86	60.6
Coog	MH051250	Mycobacterium	A5	51215	84	61.0
Cuco	JN408459	Mycobacterium	A5	50965	86	60.9
Dublin	MH338235	Mycobacterium	A5	50280	87	60.9
ElTiger69	JX042578	Mycobacterium	A5	51505	92	59.8
ForGetIt	MG099944	Mycobacterium	A5	51050	87	60.6
George	JF704107	Mycobacterium	A5	51578	82	61.0
Jabiru	MH051254	Mycobacterium	A5	50530	86	60.0
Jovo	KF493882	Mycobacterium	A5	51319	84	60.8
LittleCherry	KF017001	Mycobacterium	A5	50690	87	60.9
Midas2	MH051256	Mycobacterium	A5	51215	84	61.0
Naca	MH020239	Mycobacterium	A5	51594	87	59.8
Phlorence	MG099949	Mycobacterium	A5	50403	84	60.9
Swirley	KM101118	Mycobacterium	A5	49717	87	61.0
Tarynearal	MH338241	Mycobacterium	A5	51143	89	60.9
Theia	KT438501	Mycobacterium	A5	51543	87	60.8
Tiger	JQ684677	Mycobacterium	A5	50332	85	60.7
UnionJack	KT004677	Mycobacterium	A5	49158	86	60.0
Artemis2UCLA	KF560333	Mycobacterium	A6	52344	102	61.4
Blue7	JN698999	Mycobacterium	A6	52288	103	61.4
CloudWang3	KF560332	Mycobacterium	A6	52873	103	61.4
DaVinci	JF937092	Mycobacterium	A6	51547	97	61.5
EricB	JN049605	Mycobacterium	A6	51702	97	61.5
Gladiator	JF704097	Mycobacterium	A6	52213	96	61.4
GreedyLawyer	MH051253	Mycobacterium	A6	52091	88	61.5
Gruunaga	KX576638	Mycobacterium	A6	52195	101	61.4
Hammer	JF937094	Mycobacterium	A6	51889	104	61.3
Isiphiwo	KX641261	Mycobacterium	A6	51910	96	61.6
Jeffabunny	JN699019	Mycobacterium	A6	48963	92	61.6
Kazan	KU985096	Mycobacterium	A6	52160	101	61.6
Koko	MG099945	Mycobacterium	A6	52879	102	61.3
McFly	KU695582	Mycobacterium	A6	52502	99	61.5
Priamo	MH155876	Mycobacterium	A6	51633	98	61.4
SuperAwesome	MH020240	Mycobacterium	A6	52686	99	61.6
ToneTone	KX375815	Mycobacterium	A6	52044	96	61.5
VohminGhazi	KM401838	Mycobacterium	A6	52155	100	61.5
Wiks	MF668288	Mycobacterium	A6	52122	100	61.5
WunderPhul	MG099952	Mycobacterium	A6	48724	89	61.5
Zaka	KF560334	Mycobacterium	A6	52122	101	61.5
Zulu	MH779517	Mycobacterium	A6	52499	101	61.4
HINdeR	KC661275	Mycobacterium	A7	52617	84	62.8
Sheen	KP273225	Mycobacterium	A7	52927	84	63.4

Timshel	JF957060	Mycobacterium	A7	53278	85	63.1
Astro	JX015524	Mycobacterium	A8	52494	99	61.4
Dixon	MH651173	Mycobacterium	A8	52055	99	61.4
NearlyHeadless	MH825708	Mycobacterium	A8	52383	101	61.4
Saintus	JN831654	Mycobacterium	A8	49228	95	61.2
Smeadley	KT184694	Mycobacterium	A8	52392	100	61.4
Alma	JN699005	Mycobacterium	A9	53177	96	62.5
Catalina	KU613353	Mycobacterium	A9	53411	101	62.6
Conquerage	MG872835	Mycobacterium	A9	52953	96	62.5
Eidsmoe	KU716094	Mycobacterium	A9	52946	96	62.5
HortumSL17	KX585252	Mycobacterium	A9	53426	100	62.6
Myxus	KU716095	Mycobacterium	A9	53425	99	62.6
PackMan	JF704110	Mycobacterium	A9	51339	93	62.6
Phaeder	KX664447	Mycobacterium	A9	53300	99	62.6
Phonnegut	MG872840	Mycobacterium	A9	53219	99	62.6
Pioneer	KT285706	Mycobacterium	A9	53219	99	62.6
Priya	MH371122	Mycobacterium	A9	52949	96	62.5
Qobbit	KX685355	Mycobacterium	A9	52911	96	62.6
Scherzo	MG872842	Mycobacterium	A9	53355	97	62.7
FF47	JX901189	Mycobacterium	AB	47724	73	58.6
Muddy	KF024728	Mycobacterium	AB	48228	71	58.8
Cuke	MG757156	Mycobacterium	AC	68869	127	49.1
Fowlmouth	MH834613	Mycobacterium	AC	69660	120	48.7
ABU	JF704091	Mycobacterium	B1	68850	100	66.5
AltPhacts	MG962362	Mycobacterium	B1	68056	99	66.5
Apizium	KR781349	Mycobacterium	B1	68227	100	66.4
Ashraf	KY385380	Mycobacterium	B1	69082	103	66.3
Badfish	KJ194580	Mycobacterium	B1	69030	102	66.5
Banjo	MH230874	Mycobacterium	B1	68296	98	66.5
BatteryCK	MG962363	Mycobacterium	B1	68509	100	66.4
BlackStallion	KY965066	Mycobacterium	B1	68714	101	66.5
Buckeye	MH450116	Mycobacterium	B1	69174	100	66.5
Chah	FJ174694	Mycobacterium	B1	68450	104	66.5
CharlieGBrown	KX576647	Mycobacterium	B1	68604	100	66.4
CheetO	MH230875	Mycobacterium	B1	68503	98	66.5
Childish	MH371114	Mycobacterium	B1	69044	102	66.5
Chorkpop	KY676783	Mycobacterium	B1	68506	103	66.4
Chunky	MG925339	Mycobacterium	B1	68249	100	66.5
Cobra	MH051264	Mycobacterium	B1	68875	102	66.4
Colbert	GQ303259	Mycobacterium	B1	67774	100	66.5
Craff	MH399773	Mycobacterium	B1	69263	103	66.4
Crownjwl	MH779500	Mycobacterium	B1	68521	99	66.6
Daffy	KX683293	Mycobacterium	B1	68492	101	66.5
Derpp	KX576645	Mycobacterium	B1	68637	101	66.4
Dingo	MF919503	Mycobacterium	B1	67776	98	66.6
Doddsville	MH371107	Mycobacterium	B1	68243	98	66.4
DoesntMatter	MG962365	Mycobacterium	B1	68331	99	66.5
DonSanchon	MH576970	Mycobacterium	B1	68015	97	66.5
DuchessDung	MH051251	Mycobacterium	B1	68235	99	66.4

EmpTee	KJ567044	Mycobacterium	B1	68428	101	66.5
Eremos	KM236502	Mycobacterium	B1	68472	99	66.5
Fang	GU247133	Mycobacterium	B1	68569	102	66.5
FluffyNinja	KP027197	Mycobacterium	B1	68716	103	66.5
FriarPreacher	KX576643	Mycobacterium	B1	68415	99	66.4
FugateOSU	MH479914	Mycobacterium	B1	69476	102	66.5
Gareth	MH399775	Mycobacterium	B1	68866	102	66.5
GeneCoco	MH479916	Mycobacterium	B1	68547	100	66.4
Gophee	MH651175	Mycobacterium	B1	68856	101	66.5
Grand2040	MH744417	Mycobacterium	B1	68591	99	66.4
Gyarad	JX649099	Mycobacterium	B1	68004	99	66.5
Haimas	MG770212	Mycobacterium	B1	68296	99	66.5
Hamish	MH825702	Mycobacterium	B1	68584	99	66.5
Harvey	JF937095	Mycobacterium	B1	68193	99	66.5
Held	KX683292	Mycobacterium	B1	68314	100	66.5
Hertubise	JF937097	Mycobacterium	B1	68675	101	66.4
Hetaeria	KT364588	Mycobacterium	B1	68405	101	66.5
HighStump	MG757158	Mycobacterium	B1	68821	104	66.5
Horchata	MF919507	Mycobacterium	B1	68171	96	66.4
HSavage	MH576954	Mycobacterium	B1	67993	99	66.5
ImtiyazSitla	KY385382	Mycobacterium	B1	69082	103	66.3
Iridoclysis	KX592589	Mycobacterium	B1	68597	101	66.4
IsaacEli	JN698990	Mycobacterium	B1	68839	104	66.5
JacAttac	JN698989	Mycobacterium	B1	68311	102	66.5
JangoPhett	MG757159	Mycobacterium	B1	68673	100	66.5
Kahve	MH371117	Mycobacterium	B1	69031	102	66.5
Kailash	MF919511	Mycobacterium	B1	68953	100	66.3
Kikipoo	JN699017	Mycobacterium	B1	68839	104	66.5
KingTut	MH450122	Mycobacterium	B1	64827	92	66.5
KingVeVeVe	KJ538723	Mycobacterium	B1	68043	100	66.5
KlimbOn	MH651177	Mycobacterium	B1	68596	101	66.5
KLucky39	JF704099	Mycobacterium	B1	68138	100	66.5
Kwksand96	MH513973	Mycobacterium	B1	68822	103	66.4
Labeouficaum	MH479918	Mycobacterium	B1	68637	102	66.4
Lasso	KM408320	Mycobacterium	B1	68576	101	66.4
LeeLot	MG925346	Mycobacterium	B1	68921	100	66.5
Lego3393	KX620786	Mycobacterium	B1	69037	102	66.5
LemonSlice	MF155947	Mycobacterium	B1	68324	102	66.5
Longacauda	MF919519	Mycobacterium	B1	68804	102	66.4
Lulumae	MF668276	Mycobacterium	B1	68056	97	66.6
Mana	KX578071	Mycobacterium	B1	68479	99	66.4
Manad	KJ595576	Mycobacterium	B1	68807	101	66.4
Maskar	KY385383	Mycobacterium	B1	69082	103	66.3
Megatron	MG925348	Mycobacterium	B1	69009	103	66.5
Mesh1	MH825705	Mycobacterium	B1	68774	102	66.4
MichaelPhcott	MH576958	Mycobacterium	B1	68494	100	66.4
Mikota	MF919523	Mycobacterium	B1	67768	96	66.6
MitKao	KX670813	Mycobacterium	B1	68493	102	66.5
Morgushi	JN638753	Mycobacterium	B1	68307	99	66.4

Morty	MH371125	Mycobacterium	B1	68878	102	66.5
Mosaic	MG925350	Mycobacterium	B1	68488	99	66.5
Mulan	MH727555	Mycobacterium	B1	68521	101	66.5
Murdoc	JN638752	Mycobacterium	B1	68600	98	66.4
Mutante	MH399780	Mycobacterium	B1	68494	103	66.5
Nacho	JX649098	Mycobacterium	B1	69321	104	66.6
Newman	KC691258	Mycobacterium	B1	68598	97	66.5
Numberten	KJ194583	Mycobacterium	B1	68607	102	66.5
Oline	JN192463	Mycobacterium	B1	68720	101	66.4
Olive	MH077582	Mycobacterium	B1	68740	98	66.5
OliverWalter	MG925356	Mycobacterium	B1	68792	101	66.5
Oosterbaan	JF704109	Mycobacterium	B1	68735	102	66.5
Orion	DQ398046	Mycobacterium	B1	68427	100	66.5
OSmaximus	JN006064	Mycobacterium	B1	69118	102	66.3
PG1	AF547430	Mycobacterium	B1	68999	100	66.5
Phamished	KR816508	Mycobacterium	B1	68515	101	66.5
Phareon	MH651184	Mycobacterium	B1	68040	99	66.5
PhatCats2014	KX369585	Mycobacterium	B1	69000	101	66.5
PhenghisKhan	MG757164	Mycobacterium	B1	68727	102	66.3
Phergie	MG757165	Mycobacterium	B1	68716	102	66.3
Phipps	JF704102	Mycobacterium	B1	68293	99	66.5
Phleuron	MH316568	Mycobacterium	B1	68527	99	66.4
PhrankReynolds	MH155874	Mycobacterium	B1	68721	100	66.3
PhrodoBaggins	MH399786	Mycobacterium	B1	68873	104	66.4
Phunky	MF919528	Mycobacterium	B1	68375	99	66.5
Piglet	JX649097	Mycobacterium	B1	68992	101	66.5
PinheadLarry	MH590594	Mycobacterium	B1	68390	100	66.4
Pinkman	KX702319	Mycobacterium	B1	68938	101	66.4
Pipsqueak	KP027208	Mycobacterium	B1	68328	99	66.5
Placalicious	MH479921	Mycobacterium	B1	69085	99	66.5
Podrick	MH651186	Mycobacterium	B1	68406	100	66.4
Pops	KR997967	Mycobacterium	B1	68367	99	66.6
Potter	KU867907	Mycobacterium	B1	68327	96	66.5
ProfessorX	MG962375	Mycobacterium	B1	68086	99	66.5
Puhltonio	GQ303264	Mycobacterium	B1	68323	97	66.4
Roy17	MH513980	Mycobacterium	B1	68056	99	66.6
Scoot17C	GU247134	Mycobacterium	B1	68432	102	66.5
SDcharge11	KC661274	Mycobacterium	B1	67702	100	66.5
Serendipity	JN006063	Mycobacterium	B1	68804	101	66.5
Serpentine	JX649096	Mycobacterium	B1	68884	104	66.5
Sheila	MF919530	Mycobacterium	B1	67880	96	66.5
ShiVal	KC576784	Mycobacterium	B1	68355	101	66.5
Sigman	KP027209	Mycobacterium	B1	68311	101	66.5
Soto	KJ174157	Mycobacterium	B1	67744	98	66.6
Squid	KT599441	Mycobacterium	B1	68596	101	66.5
Suffolk	KF713485	Mycobacterium	B1	68262	97	66.6
Swish	KJ194579	Mycobacterium	B1	68735	103	66.5
TallGrassMM	JN699010	Mycobacterium	B1	68133	100	66.5
Thora	JF957056	Mycobacterium	B1	68839	100	66.5

ThreeOh3D2	JN699009	Mycobacterium	B1	68992	103	66.5
Trypo	MG944223	Mycobacterium	B1	68798	101	66.4
TyrionL	KX576646	Mycobacterium	B1	68637	102	66.4
UAch1	MH576967	Mycobacterium	B1	68405	102	66.4
UncleHowie	GQ303266	Mycobacterium	B1	68016	98	66.5
Vaticameos	MH590588	Mycobacterium	B1	66887	99	66.5
Virapocalypse	MF919539	Mycobacterium	B1	68682	103	66.5
Vista	JN699008	Mycobacterium	B1	68494	101	66.5
Vivaldi	KM347890	Mycobacterium	B1	68873	102	66.4
Vortex	JF704103	Mycobacterium	B1	68346	99	66.5
Waterdiva	MH779516	Mycobacterium	B1	68866	103	66.5
Xavier	MG944225	Mycobacterium	B1	68493	101	66.4
Yoshand	JF937109	Mycobacterium	B1	68719	103	66.5
Zonia	KM363597	Mycobacterium	B1	69271	102	66.3
Arbiter	JN618996	Mycobacterium	B2	67169	88	68.9
Ares	JN699004	Mycobacterium	B2	67436	92	69.0
Boyle	MH051249	Mycobacterium	B2	67491	92	69.0
Eaglehorse	MH727546	Mycobacterium	B2	67391	93	69.0
FrenchFry	MH697584	Mycobacterium	B2	67494	93	68.9
Glass	KT880194	Mycobacterium	B2	67509	94	69.0
Godines	KR997932	Mycobacterium	B2	67277	91	69.0
Hedgerow	JN698991	Mycobacterium	B2	67451	92	69.0
Holeinone	MG812490	Mycobacterium	B2	67044	91	68.9
ItsyBitsy1	MG812491	Mycobacterium	B2	67570	89	68.9
Kheth	MH001452	Mycobacterium	B2	67406	92	68.9
Laurie	KX443696	Mycobacterium	B2	66507	90	69.0
LizLemon	KM101117	Mycobacterium	B2	67496	92	68.9
Opia	MG757162	Mycobacterium	B2	67401	93	68.9
Qyrzula	DQ398048	Mycobacterium	B2	67188	81	69.0
Rosebush	AY129334	Mycobacterium	B2	67480	90	69.0
Sabella	MH590591	Mycobacterium	B2	67304	92	68.9
TA17a	KF024722	Mycobacterium	B2	67324	95	69.0
Tres	KT365402	Mycobacterium	B2	67349	92	68.9
Akoma	JN699006	Mycobacterium	B3	68711	104	67.5
Athena	JN699003	Mycobacterium	B3	69409	105	67.5
Audrey	KJ194581	Mycobacterium	B3	68662	103	67.5
Baloo	MG920059	Mycobacterium	B3	68514	102	67.5
Bernardo	KF493879	Mycobacterium	B3	68196	100	67.4
ChaChing	MG925338	Mycobacterium	B3	68668	103	67.5
Chandler	KP027207	Mycobacterium	B3	69451	103	67.5
Corofin	KR080205	Mycobacterium	B3	68685	103	67.5
Daisy	JF704095	Mycobacterium	B3	68245	102	67.6
Gadjet	JN698992	Mycobacterium	B3	67949	102	67.5
Heathcliff	KJ194584	Mycobacterium	B3	68628	103	67.5
Kamiyu	JN699018	Mycobacterium	B3	68633	103	67.5
Mortcellus	MH316565	Mycobacterium	B3	69800	103	67.5
Morty007	MH513976	Mycobacterium	B3	69431	101	67.5
Nozo	MG925353	Mycobacterium	B3	69439	103	67.5
OrangeOswald	KR080203	Mycobacterium	B3	68674	102	67.5

Phaedrus	EU816589	Mycobacterium	B3	68090	98	67.6
Phlyer	FJ641182	Mycobacterium	B3	69378	103	67.5
Pipefish	DQ398049	Mycobacterium	B3	69059	102	67.3
RagingRooster	MG839014	Mycobacterium	B3	68670	103	67.6
Tydolla	MH576977	Mycobacterium	B3	68657	99	67.5
Yahalom	MH051265	Mycobacterium	B3	68482	101	67.4
AlanGrant	KR080200	Mycobacterium	B4	72109	99	68.9
BrownCNA	KT270441	Mycobacterium	B4	71214	97	68.9
ChrisnMich	JF704094	Mycobacterium	B4	70428	100	69.1
Cooper	DQ398044	Mycobacterium	B4	70654	99	69.1
Fortunato	KX589269	Mycobacterium	B4	70679	94	69.0
Hangman	MH513970	Mycobacterium	B4	71376	97	68.9
JAMaL	KF493881	Mycobacterium	B4	70841	98	68.8
Nigel	EU770221	Mycobacterium	B4	69904	94	68.3
Stinger	JN699011	Mycobacterium	B4	69641	95	68.6
Vincenzo	KR080194	Mycobacterium	B4	72139	98	68.9
Zemanar	JF704104	Mycobacterium	B4	71092	95	68.9
Acadian	JN699007	Mycobacterium	B5	69864	97	68.4
Baee	KR080199	Mycobacterium	B5	70270	96	67.6
Phelemich	KF416341	Mycobacterium	B5	70115	95	68.3
Reprobate	KF024727	Mycobacterium	B5	70120	97	68.3
Rich	KY224000	Mycobacterium	B5	69819	95	67.6
Serendipitous	MH727561	Mycobacterium	B5	69867	98	68.0
39HC	KJ433973	Mycobacterium	B6	71565	100	70.0
40BC	KJ433975	Mycobacterium	B6	71565	100	70.0
Hosp	KJ433974	Mycobacterium	B6	70667	97	69.9
Jolie1	KJ433976	Mycobacterium	B6	71058	98	69.9
KayaCho	KF024729	Mycobacterium	B6	70838	95	70.0
Saguaro	MH744423	Mycobacterium	B7	69448	93	69.5
Thonko	MH632120	Mycobacterium	B8	69471	108	68.6
Alice	JF704092	Mycobacterium	C1	153401	222	64.7
ArcherS7	KC748970	Mycobacterium	C1	156558	237	64.7
Astraea	KC691257	Mycobacterium	C1	154872	232	64.7
Audrick	MF919493	Mycobacterium	C1	155205	235	64.7
Ava3	JQ911768	Mycobacterium	C1	154466	232	64.8
Bangla1971	MH825697	Mycobacterium	C1	154722	235	64.7
BeanWater	MF919494	Mycobacterium	C1	154061	225	64.7
Bigswole	MF919495	Mycobacterium	C1	156514	237	64.8
Bread	MH779498	Mycobacterium	C1	153796	231	64.8
Breeniome	KF006817	Mycobacterium	C1	154434	236	64.8
Bxz1	AY129337	Mycobacterium	C1	156102	225	64.8
Cali	EU826471	Mycobacterium	C1	155372	222	64.7
Cane17	MH697579	Mycobacterium	C1	160330	223	64.6
Catera	DQ398053	Mycobacterium	C1	153766	218	64.7
CharlieB	MH727543	Mycobacterium	C1	155886	232	64.7
Daffodil	MF919499	Mycobacterium	C1	155034	238	64.7
Dandelion	JN412588	Mycobacterium	C1	157568	238	64.7
Derek	MH316561	Mycobacterium	C1	156199	229	64.7
Drazdys	JF704116	Mycobacterium	C1	156281	230	64.7

DTDevon	KT365398	Mycobacterium	C1	156754	233	64.6
Erdmann	KX721256	Mycobacterium	C1	155565	233	64.7
ErnieJ	KT365400	Mycobacterium	C1	153243	226	64.7
ET08	GQ303260	Mycobacterium	C1	155445	218	64.6
FudgeTart	MH779502	Mycobacterium	C1	154658	232	64.8
Gabriel	KX781992	Mycobacterium	C1	154474	233	64.8
Ghost	JF704096	Mycobacterium	C1	155167	231	64.6
Gizmo	KC748968	Mycobacterium	C1	157482	241	64.6
HyRo	KT281790	Mycobacterium	C1	153714	224	64.7
InigoMontoya	MH697586	Mycobacterium	C1	155217	237	64.7
InterFolia	MH051260	Mycobacterium	C1	156221	237	64.7
Koguma	MF919513	Mycobacterium	C1	155759	230	64.7
LifeSavor	MG872839	Mycobacterium	C1	156804	233	64.6
LinStu	JN412592	Mycobacterium	C1	153882	226	64.8
Littleton	KX369583	Mycobacterium	C1	155800	236	64.7
LRRHood	GQ303262	Mycobacterium	C1	154349	224	64.7
Lukilu	KX831080	Mycobacterium	C1	157034	221	64.7
Megamind	MH669008	Mycobacterium	C1	154780	230	64.8
MikeLiesIn	MH051266	Mycobacterium	C1	155916	234	64.7
Momo	KR080196	Mycobacterium	C1	154553	232	64.7
MoMoMixon	JN699626	Mycobacterium	C1	154573	228	64.8
Nappy	JN699627	Mycobacterium	C1	156646	232	64.7
NuevoMundo	MG812493	Mycobacterium	C1	155943	236	64.7
ParkTD	MH316567	Mycobacterium	C1	155006	234	64.8
Phlegm	MH697590	Mycobacterium	C1	155959	238	64.7
Phox	MF919527	Mycobacterium	C1	154874	229	64.7
Pier	MG812494	Mycobacterium	C1	156471	234	64.7
Pio	JN699013	Mycobacterium	C1	156758	238	64.8
Pleione	JN624850	Mycobacterium	C1	155586	236	64.7
Rabinovish	MH590593	Mycobacterium	C1	154861	229	64.7
Rizal	EU826467	Mycobacterium	C1	153894	220	64.7
Roots515	MH744422	Mycobacterium	C1	156288	237	64.7
Salacia	MH727558	Mycobacterium	C1	156610	237	64.7
ScottMcG	EU826469	Mycobacterium	C1	154017	221	64.8
Sebata	JN204348	Mycobacterium	C1	155286	230	64.8
Shrimp	KF024734	Mycobacterium	C1	155714	235	64.7
Spud	EU826468	Mycobacterium	C1	154906	222	64.8
Tonenili	KX752698	Mycobacterium	C1	160985	258	64.1
Tyke	MH051261	Mycobacterium	C1	156679	231	64.7
Wally	JN699625	Mycobacterium	C1	155299	228	64.7
Willis	KJ595575	Mycobacterium	C1	155476	233	64.7
Yucca	KX721255	Mycobacterium	C1	155582	232	64.7
Zalkecks	MH825713	Mycobacterium	C1	154764	234	64.7
Zeenon	KT321476	Mycobacterium	C1	155292	232	64.7
ZygoTaiga	KM881426	Mycobacterium	C1	157204	231	64.7
Myrna	EU826466	Mycobacterium	C2	164602	229	65.4
Phabba	MF668280	Mycobacterium	C2	164254	244	65.2
Adjutor	EU676000	Mycobacterium	D1	64511	86	59.7
BigMama	MH025888	Mycobacterium	D1	64592	87	59.7

Butterscotch	FJ168660	Mycobacterium	D1	64562	86	59.7
Erk16	MH316562	Mycobacterium	D1	64877	89	59.6
Gumball	FJ168661	Mycobacterium	D1	64807	88	59.6
KandZ	MH834615	Mycobacterium	D1	64596	86	59.7
Nova	JN699014	Mycobacterium	D1	65108	88	59.7
PBI1	DQ398047	Mycobacterium	D1	64494	81	59.7
PLot	DQ398051	Mycobacterium	D1	64787	89	59.7
SirHarley	JF937107	Mycobacterium	D1	64791	90	59.6
Troll4	FJ168662	Mycobacterium	D1	64618	88	59.6
Visconti	MH399788	Mycobacterium	D1	64570	89	59.7
Hawkeye	KJ194582	Mycobacterium	D2	67383	104	57.1
244	DQ398041	Mycobacterium	E	74483	142	62.9
ABCat	KF188414	Mycobacterium	E	76131	143	63.0
Adnama	MH371112	Mycobacterium	E	75208	148	62.9
Asriel	MG872831	Mycobacterium	E	74594	142	62.8
Barbarian	MG872832	Mycobacterium	E	74594	144	62.8
Bask21	JF937091	Mycobacterium	E	74997	147	62.9
Bruin	KF562099	Mycobacterium	E	74210	141	63.0
Cjw1	AY129331	Mycobacterium	E	75931	141	63.1
Contagion	KF024732	Mycobacterium	E	74533	140	63.1
CrystalP	KY319168	Mycobacterium	E	76483	143	63.0
DoctorDiddles	MH399774	Mycobacterium	E	75070	143	62.8
DrDrey	KF306380	Mycobacterium	E	77367	144	63.0
Dumbo	KC691255	Mycobacterium	E	75736	147	63.0
Dusk	KT222942	Mycobacterium	E	75339	142	63.0
Easy2Say	MH651174	Mycobacterium	E	75598	146	62.9
Elph10	JN391441	Mycobacterium	E	74675	143	63.0
Emmina	MH669002	Mycobacterium	E	75295	146	62.9
Eureka	JN412590	Mycobacterium	E	76174	145	62.9
FireRed	MF919506	Mycobacterium	E	76217	148	63.0
Gage	MG872837	Mycobacterium	E	71650	135	63.2
Gemini	MH779503	Mycobacterium	E	75649	149	62.9
Glexan	MH536820	Mycobacterium	E	76498	148	62.9
Goku	KF416343	Mycobacterium	E	76483	143	62.8
Henry	JF937096	Mycobacterium	E	76049	143	63.0
Hopey	MH576953	Mycobacterium	E	75586	142	63.0
HufflyPuff	KF562100	Mycobacterium	E	76323	146	63.1
Icee	MH399778	Mycobacterium	E	74169	144	63.0
IHOP	MH513972	Mycobacterium	E	75668	149	62.9
Inca	MH576956	Mycobacterium	E	74070	145	62.8
Kimchi	MG757160	Mycobacterium	E	75829	149	62.9
Kostya	EU816591	Mycobacterium	E	75811	143	62.9
Lilac	JN382248	Mycobacterium	E	76260	140	63.0
MadamMonkfish	MF668277	Mycobacterium	E	75554	146	63.0
Maxxinista	KY549152	Mycobacterium	E	75215	146	63.0
Mindy	KR080204	Mycobacterium	E	75796	147	63.0
MISSy	MF919524	Mycobacterium	E	75808	145	63.1
Mosby	KF493883	Mycobacterium	E	74533	141	63.1
MPhalcon	MH020247	Mycobacterium	E	75605	146	63.1

Murica	MF919525	Mycobacterium	E	77053	147	63.0
Murphy	KC748971	Mycobacterium	E	76179	146	62.9
Nala	KF562101	Mycobacterium	E	75894	147	63.1
NelitzaMV	KT222941	Mycobacterium	E	72790	139	63.1
NoSleep	KT020852	Mycobacterium	E	74655	143	63.0
Paperbeatsrock	MH727557	Mycobacterium	E	75640	142	63.0
Phaja	MH513978	Mycobacterium	E	75685	149	62.9
Pharsalus	KX611831	Mycobacterium	E	75779	142	63.0
PhatBacter	KF562102	Mycobacterium	E	76217	148	63.0
Phaux	KC748969	Mycobacterium	E	76479	145	62.9
Phrux	KC661277	Mycobacterium	E	74711	141	63.1
Porky	EU816588	Mycobacterium	E	76312	147	62.8
Pumpkin	GQ303265	Mycobacterium	E	74491	143	63.0
Rakim	JN006062	Mycobacterium	E	75706	145	62.9
RiverMonster	MH000607	Mycobacterium	E	75565	144	63.1
Sassay	MF919529	Mycobacterium	E	73495	139	63.0
ShereKhan	MH513983	Mycobacterium	E	76133	143	63.1
Simpliphy	MH536827	Mycobacterium	E	75366	145	63.0
SirDuracell	JF937106	Mycobacterium	E	75973	145	62.9
Sotrice96	MG872843	Mycobacterium	E	76299	149	63.1
TBrady12	MH536829	Mycobacterium	E	75359	149	62.8
TeardropMSU	KU865303	Mycobacterium	E	74896	143	63.0
Terminus	MF919535	Mycobacterium	E	76169	147	63.1
Toto	JN006061	Mycobacterium	E	75933	140	63.0
Tuco	KX817173	Mycobacterium	E	76944	148	63.0
Ukulele	KT373978	Mycobacterium	E	75114	141	63.0
Willez	MF919540	Mycobacterium	E	74576	142	62.9
xkcd	MH590587	Mycobacterium	E	76915	149	62.8
YassJohnny	MF919541	Mycobacterium	E	73697	139	62.9
Youngblood	MG099953	Mycobacterium	E	75896	148	62.9
Renaud18	MH651187	Mycobacterium	F	58078	112	61.7
Alexphander	MG962361	Mycobacterium	F1	57734	104	61.2
ArcusAngelus	MH744415	Mycobacterium	F1	58569	104	61.4
Ardmore	GU060500	Mycobacterium	F1	52141	87	61.5
Batiatus	MH020235	Mycobacterium	F1	57737	105	61.8
BigPhil	MH051248	Mycobacterium	F1	53618	100	61.5
Bipolar	KM597530	Mycobacterium	F1	58985	106	61.4
BobaPhett	MH155865	Mycobacterium	F1	59815	108	61.3
Bobi	KF114874	Mycobacterium	F1	59179	107	61.7
Boomer	EU816590	Mycobacterium	F1	58037	105	61.1
Brocalys	KU865302	Mycobacterium	F1	54751	96	61.8
Bubbles123	KY348865	Mycobacterium	F1	57647	100	61.4
Burwell21	MH651169	Mycobacterium	F1	58098	100	61.5
BuzzLyseyear	KM347889	Mycobacterium	F1	59419	110	61.1
ByChance	MH399771	Mycobacterium	F1	53123	95	61.3
Byougenkin	MH155866	Mycobacterium	F1	54685	101	61.4
Cabrinians	KT895281	Mycobacterium	F1	56669	101	61.2
CaptainTrips	KM652553	Mycobacterium	F1	57328	107	61.5
Cerasum	KM215148	Mycobacterium	F1	53636	102	61.5

Che8	AY129330	Mycobacterium	F1	59471	112	61.3
Clifton	MH371115	Mycobacterium	F1	55727	96	61.7
Daenerys	KF017005	Mycobacterium	F1	58043	102	61.6
Dante	KT309034	Mycobacterium	F1	59652	105	61.8
DaWorst	MF919501	Mycobacterium	F1	56827	103	61.0
DeadP	JN698996	Mycobacterium	F1	56461	106	61.6
DillTech15	MH077578	Mycobacterium	F1	57797	111	61.9
DLane	JF937093	Mycobacterium	F1	58899	105	61.9
Dorothy	JX411620	Mycobacterium	F1	58866	104	61.4
DotProduct	JN859129	Mycobacterium	F1	55363	98	61.8
Drago	JN542517	Mycobacterium	F1	54411	103	61.2
EleanorGeorge	MH669001	Mycobacterium	F1	59482	108	61.7
Emma	MF668270	Mycobacterium	F1	56418	109	61.3
Empress	KY012363	Mycobacterium	F1	57155	104	61.5
Estave1	KM279937	Mycobacterium	F1	60727	112	61.3
Florinda	KR997930	Mycobacterium	F1	59416	117	61.7
Frankie	MG812488	Mycobacterium	F1	57036	103	61.9
Fruitloop	FJ174690	Mycobacterium	F1	58471	102	61.8
Galactic	MH727548	Mycobacterium	F1	57861	109	61.4
Geralt	MF668271	Mycobacterium	F1	55765	97	61.5
Girafales	KR997931	Mycobacterium	F1	58456	112	61.7
Girr	MH669003	Mycobacterium	F1	57754	102	61.4
Gorge	MH590602	Mycobacterium	F1	57955	103	61.4
GUmbie	JN398368	Mycobacterium	F1	57387	104	61.4
Hades	KM101122	Mycobacterium	F1	54986	102	61.4
Hamulus	KF024723	Mycobacterium	F1	57155	105	61.8
Harley	MH632119	Mycobacterium	F1	58731	107	61.4
Ibhubesi	JF937098	Mycobacterium	F1	55600	103	61.2
Inventum	KM066034	Mycobacterium	F1	57052	102	61.4
Job42	KC661280	Mycobacterium	F1	59626	106	61.2
JoeyJr	MH669005	Mycobacterium	F1	58962	107	61.4
Kersh	KX610764	Mycobacterium	F1	60190	107	61.2
Kimberlium	KR935214	Mycobacterium	F1	56826	105	61.4
Kingsley	KY702574	Mycobacterium	F1	60300	112	61.4
Koella	MH316564	Mycobacterium	F1	54118	95	61.5
Krakatau	MH590598	Mycobacterium	F1	53058	97	61.5
KristaRAM	MH651178	Mycobacterium	F1	58701	113	61.6
Lizziana	MH834617	Mycobacterium	F1	58423	105	61.3
Llama	KM402757	Mycobacterium	F1	58472	111	61.1
Llij	DQ398045	Mycobacterium	F1	56852	100	61.5
Mantra	MH590596	Mycobacterium	F1	57001	102	61.5
Mattes	MH155871	Mycobacterium	F1	58074	105	61.3
Melissauren88	MH077580	Mycobacterium	F1	54356	95	61.8
MilleniumForce	MH825707	Mycobacterium	F1	58106	104	61.8
Misha28	MH020242	Mycobacterium	F1	57455	93	62.9
Mozy	JF937102	Mycobacterium	F1	57278	108	61.1
Mutaforma13	JN020142	Mycobacterium	F1	57701	106	61.3
Nimbo	MH669009	Mycobacterium	F1	55767	96	61.5
Nivrat	MH651183	Mycobacterium	F1	58009	103	61.5

NormanBulbieJr	MH399784	Mycobacterium	F1	58102	102	61.3
Ogopogo	MG925354	Mycobacterium	F1	56867	110	61.1
OldBen	MG770213	Mycobacterium	F1	57159	101	61.5
OlympiaSaint	MH371120	Mycobacterium	F1	58297	107	61.4
Ovechkin	KR824843	Mycobacterium	F1	58338	106	62.0
OwlsT2W	MH051257	Mycobacterium	F1	56515	103	61.2
Pacc40	FJ174692	Mycobacterium	F1	58554	101	61.3
PHappiness	MH669010	Mycobacterium	F1	57989	104	61.6
Phasih	MF919526	Mycobacterium	F1	56125	100	61.1
Phatniss	KT279576	Mycobacterium	F1	57293	101	61.3
Pippy	KY965064	Mycobacterium	F1	56663	103	61.5
PMC	DQ398050	Mycobacterium	F1	56692	104	61.4
PopTart	KT281792	Mycobacterium	F1	55094	95	61.6
Priscilla	MG872841	Mycobacterium	F1	58230	105	61.3
QuickMath	MH669012	Mycobacterium	F1	57526	105	61.7
Quico	KR997968	Mycobacterium	F1	58671	112	61.7
Ramsey	FJ174693	Mycobacterium	F1	58578	108	61.2
RitaG	MF668281	Mycobacterium	F1	57789	103	61.6
RockyHorror	JF704117	Mycobacterium	F1	56719	107	61.1
Ruby	MH651188	Mycobacterium	F1	57726	99	61.4
Saal	KJ025956	Mycobacterium	F1	57775	101	61.3
Sandalphon	MH371123	Mycobacterium	F1	59540	104	61.4
SassyB	KY471267	Mycobacterium	F1	55094	95	61.6
Seagreen	KT281793	Mycobacterium	F1	57766	105	61.8
SG4	JN699012	Mycobacterium	F1	59016	105	61.9
Shauna1	JN020141	Mycobacterium	F1	59315	107	61.7
ShiLan	JN020143	Mycobacterium	F1	59794	107	61.4
SimranZ1	KY385384	Mycobacterium	F1	56335	103	61.3
SiSi	KC661278	Mycobacterium	F1	56279	99	61.5
Sparkdehlily	KT895280	Mycobacterium	F1	56275	111	61.2
Spartacus	JQ300538	Mycobacterium	F1	61164	110	61.7
Spikelee	MH669014	Mycobacterium	F1	58604	104	61.0
Spoonbill	MF190168	Mycobacterium	F1	55735	103	61.3
SuperGrey	KX808131	Mycobacterium	F1	59346	103	61.7
Taj	JX121091	Mycobacterium	F1	58550	110	61.9
TootsiePop	MH020243	Mycobacterium	F1	57455	93	62.9
Tweety	EF536069	Mycobacterium	F1	58692	109	61.7
UncleRicky	MH001448	Mycobacterium	F1	57256	105	61.3
Velveteen	KF017004	Mycobacterium	F1	54314	100	61.5
Wachhund	MF668287	Mycobacterium	F1	54513	101	61.7
Wee	HQ728524	Mycobacterium	F1	59230	109	61.8
WillSterrel	KX576644	Mycobacterium	F1	58608	104	61.6
XFactor	KT281795	Mycobacterium	F1	55617	95	61.7
Avani	JQ809702	Mycobacterium	F2	54470	107	61.0
Che9d	AY129336	Mycobacterium	F2	56275	111	60.9
Demsculpinboyz	MF919502	Mycobacterium	F2	57437	117	60.9
Jabbawokkie	KF017003	Mycobacterium	F2	55213	106	61.1
Yoshi	JF704115	Mycobacterium	F2	58714	116	61.0
Zapner	KJ567041	Mycobacterium	F2	55307	108	61.1

Squirty	KM101124	Mycobacterium	F3	60285	102	62.4
TChen	MH077585	Mycobacterium	F4	57742	101	62.3
Angel	FJ973624	Mycobacterium	G1	41441	61	66.7
Annihilator	KT365399	Mycobacterium	G1	41893	62	66.6
Aroostook	MF668268	Mycobacterium	G1	41903	63	66.6
Avrafan	JN699002	Mycobacterium	G1	41901	62	66.6
BPs	EU568876	Mycobacterium	G1	41901	63	66.6
BruceB	KX443326	Mycobacterium	G1	41901	63	66.6
Cedasite	KT355472	Mycobacterium	G1	41901	63	66.6
Chance64	MF919497	Mycobacterium	G1	41903	63	66.6
Cherrybomb426	MH590605	Mycobacterium	G1	41456	62	66.7
CLED96	MH001456	Mycobacterium	G1	41456	62	66.7
DMoney	MH371116	Mycobacterium	G1	41880	61	66.6
Frosty24	KT355474	Mycobacterium	G1	41901	63	66.6
Gideon	MF668272	Mycobacterium	G1	41903	63	66.6
Gomashi	KM923970	Mycobacterium	G1	41903	62	66.6
Grizzly	MH779505	Mycobacterium	G1	41752	62	66.8
Halo	DQ398042	Mycobacterium	G1	42289	64	66.7
Hope	GQ303261	Mycobacterium	G1	41901	63	66.6
Hotshotbaby7	MH779507	Mycobacterium	G1	41903	63	66.6
Jane	KX588251	Mycobacterium	G1	41901	63	66.6
Kasen3	MH779509	Mycobacterium	G1	41890	64	66.6
Liefie	JN412593	Mycobacterium	G1	41650	61	66.8
LouisV14	MG099946	Mycobacterium	G1	44145	65	66.6
Mowgli	MH479920	Mycobacterium	G1	41902	63	66.6
OctaviousRex	MH371119	Mycobacterium	G1	41880	62	66.6
Olga	MH779513	Mycobacterium	G1	41902	64	66.6
Paito	MH779514	Mycobacterium	G1	42311	68	66.0
Phish	MH077584	Mycobacterium	G1	41901	62	66.6
Phreak	KT347314	Mycobacterium	G1	41901	63	66.6
Plagueis	MH450127	Mycobacterium	G1	41722	63	66.6
Remy19	MH001455	Mycobacterium	G1	41901	63	66.6
Schiebel	MH045569	Mycobacterium	G1	41441	61	66.7
Sneeze	KX534004	Mycobacterium	G1	42429	64	66.5
Sweets	MH779515	Mycobacterium	G1	41896	63	66.6
Taheera	KX641265	Mycobacterium	G1	41077	60	66.4
Terror	KX641266	Mycobacterium	G1	41077	60	66.4
Zombie	KX664455	Mycobacterium	G1	41901	63	66.6
Avocado	MF141540	Mycobacterium	G2	45389	68	68.7
Cambiare	KR080198	Mycobacterium	G2	45161	67	68.8
FlagStaff	KR080197	Mycobacterium	G2	44576	65	68.5
MOOREtheMARYer	KR080202	Mycobacterium	G3	44492	67	68.6
Jolie2	KJ410133	Mycobacterium	G4	44306	62	68.1
Cborch11	MH509445	Mycobacterium	H1	68508	94	57.6
Damien	KJ567046	Mycobacterium	H1	68386	93	57.6
Konstantine	FJ174691	Mycobacterium	H1	68952	95	57.3
Oaker	KF986247	Mycobacterium	H1	69099	92	57.5
Predator	EU770222	Mycobacterium	H1	70110	92	56.3
Thumb	MG962376	Mycobacterium	H1	68408	94	57.5

Barnyard	AY129339	Mycobacterium	H2	70797	109	57.3
Babsiella	JN699001	Mycobacterium	I1	48420	78	67.1
Brujita	FJ168659	Mycobacterium	I1	47057	74	66.8
Island3	HM152765	Mycobacterium	I1	47287	76	66.8
Che9c	AY129333	Mycobacterium	I2	57050	84	65.4
Sbash	KP027201	Mycobacterium	I2	55832	89	65.6
Ariel	KM400683	Mycobacterium	J	109801	245	61.0
BAKA	JF937090	Mycobacterium	J	111688	242	60.7
Constella	MH399772	Mycobacterium	J	110169	228	60.8
Courthouse	JN698997	Mycobacterium	J	110569	241	60.9
DmpstrDiver	MF919504	Mycobacterium	J	112285	241	60.6
Ejimix	MH697582	Mycobacterium	J	111924	234	60.9
EricMillard	MH697583	Mycobacterium	J	113536	239	60.9
Halley	MH077579	Mycobacterium	J	112351	245	60.8
Kalah2	MH727551	Mycobacterium	J	110713	230	61.0
Klein	MF919512	Mycobacterium	J	112329	248	60.8
LittleE	JF937101	Mycobacterium	J	109086	229	61.3
Lucky2013	MF133445	Mycobacterium	J	108627	237	61.1
MiaZeal	KM925136	Mycobacterium	J	110764	250	61.2
Minerva	KM101123	Mycobacterium	J	109871	238	60.7
Omega	AY129338	Mycobacterium	J	110865	237	61.4
Optimus	JF957059	Mycobacterium	J	109270	230	60.8
Porcelain	MF072690	Mycobacterium	J	109575	241	61.2
Redno2	KF114875	Mycobacterium	J	108297	231	60.9
Squint	MF668284	Mycobacterium	J	110240	238	61.0
Superphikiman	MF919534	Mycobacterium	J	109799	239	61.0
Thibault	JN201525	Mycobacterium	J	106327	216	60.8
Wanda	KF006818	Mycobacterium	J	109960	240	60.8
Zelink	MH669017	Mycobacterium	J	111740	236	60.8
ActinUp	MH051246	Mycobacterium	K1	59812	96	66.6
Adephagia	JF704105	Mycobacterium	K1	59646	94	66.6
Adonis	MH001453	Mycobacterium	K1	60031	95	66.7
AlishaPH	MH077577	Mycobacterium	K1	57034	88	66.6
Amelie	KX808132	Mycobacterium	K1	56439	77	67.1
Anaya	JF704106	Mycobacterium	K1	60835	98	66.4
Angelica	HM152764	Mycobacterium	K1	59598	94	66.4
Apocalypse	MF668267	Mycobacterium	K1	59947	98	66.4
BarrelRoll	JN643714	Mycobacterium	K1	59672	95	66.6
BEEST	MH509444	Mycobacterium	K1	59906	95	66.6
Beezoo	MH371113	Mycobacterium	K1	60494	99	66.5
Bella96	MF377440	Mycobacterium	K1	60746	97	66.1
Belladonna	MH697578	Mycobacterium	K1	59708	96	66.6
Biglebops	MH399770	Mycobacterium	K1	56454	77	67.1
CREW	KY380102	Mycobacterium	K1	59707	94	66.6
CrimD	HM152767	Mycobacterium	K1	59798	95	66.9
Dalmuri	MH727544	Mycobacterium	K1	59708	96	66.5
Deby	MG962364	Mycobacterium	K1	60463	95	66.5
DrHayes	KX657795	Mycobacterium	K1	60526	97	66.2
Emerson	KJ567045	Mycobacterium	K1	60310	100	66.6

Enkosi	KT281789	Mycobacterium	K1	59052	79	67.2
HedwigODU	KX585253	Mycobacterium	K1	59812	95	66.6
Homura	MH536821	Mycobacterium	K1	59708	94	66.6
JAWS	JN185608	Mycobacterium	K1	59749	94	66.6
Jeckyll	MF140412	Mycobacterium	K1	59708	96	66.6
Joy99	MH536822	Mycobacterium	K1	59837	97	66.6
LastHope	MF140416	Mycobacterium	K1	60934	102	66.9
LaterM	MG962371	Mycobacterium	K1	60143	95	66.5
LilPharaoh	MF919518	Mycobacterium	K1	56167	78	67.1
LindNT	KX641264	Mycobacterium	K1	60053	93	66.8
Murucutumbu	KM677211	Mycobacterium	K1	60609	95	66.7
Mynx	MH513977	Mycobacterium	K1	60055	96	66.6
Peanam	MF185722	Mycobacterium	K1	61041	98	68.5
SamuelLPlaqson	KX657794	Mycobacterium	K1	60526	97	66.2
SgtBeansprout	MH020245	Mycobacterium	K1	56439	78	67.1
Slimphazie	MF140428	Mycobacterium	K1	60143	98	66.6
Sulley	MF919532	Mycobacterium	K1	59873	94	66.4
Tachez	MF140430	Mycobacterium	K1	59556	96	66.5
TreyKay	MF472892	Mycobacterium	K1	60311	97	66.8
Urkel	KX657796	Mycobacterium	K1	60526	97	66.2
Validus	KF713486	Mycobacterium	K1	62466	105	68.4
BoostSeason	MH834601	Mycobacterium	K2	58078	94	68.2
DismalFunk	MF140408	Mycobacterium	K2	58129	94	68.3
DismalStressor	MH727545	Mycobacterium	K2	58129	94	68.3
Findley	MF140411	Mycobacterium	K2	58150	94	68.3
Marcoliusprime	KX688047	Mycobacterium	K2	58129	92	68.2
Milly	KP027206	Mycobacterium	K2	58211	94	68.3
Mufasa	KT591490	Mycobacterium	K2	58065	94	68.2
TM4	AF068845	Mycobacterium	K2	52797	92	68.1
ZoeJ	KJ510412	Mycobacterium	K2	57315	92	68.5
Hurricane	MF373841	Mycobacterium	K3	61318	98	67.1
Keshu	KP027199	Mycobacterium	K3	61251	101	67.3
MacnCheese	JX042579	Mycobacterium	K3	61567	99	67.3
Pixie	JF937104	Mycobacterium	K3	61147	100	67.3
ShedlockHolmes	KR080206	Mycobacterium	K3	61081	100	67.3
TBond007	KX683428	Mycobacterium	K3	61145	97	67.3
Chancellor	MF140402	Mycobacterium	K4	57697	94	68.0
Cheetobro	KJ944841	Mycobacterium	K4	57253	92	68.0
Fionnbharth	JN831653	Mycobacterium	K4	58076	94	68.0
Mitti	KY087992	Mycobacterium	K4	57895	94	68.0
SamScheppers	MH051258	Mycobacterium	K4	58351	94	67.6
Slarp	KT361920	Mycobacterium	K4	57256	92	68.0
Taquito	KX621007	Mycobacterium	K4	58390	94	67.5
Wintermute	MF140435	Mycobacterium	K4	58046	94	68.0
AlleyCat	MF185717	Mycobacterium	K5	62112	99	65.3
Collard	MH651171	Mycobacterium	K5	61395	94	65.6
Edugator	MF185719	Mycobacterium	K5	63344	92	65.3
Gengar	KX636165	Mycobacterium	K5	61626	95	65.0
Guillsminger	MF185720	Mycobacterium	K5	63153	95	65.0

Kratio	KM923971	Mycobacterium	K5	62738	99	65.7
Larva	JN243855	Mycobacterium	K5	62991	97	65.3
Leston	MH051255	Mycobacterium	K5	61808	94	64.9
OkiRoe	KJ567042	Mycobacterium	K5	62661	96	64.9
Omnicron	KM363596	Mycobacterium	K5	61511	95	64.0
Paola	MG962374	Mycobacterium	K5	61535	92	65.0
Rando14	MH697592	Mycobacterium	K5	59925	91	64.3
Thyatira	MH576966	Mycobacterium	K5	63874	96	64.6
Waterfoul	KX585251	Mycobacterium	K5	61248	94	64.9
Amgine	MF324915	Mycobacterium	K6	62236	97	66.4
Amohnition	MF140398	Mycobacterium	K6	61761	94	67.2
Cain	MF324913	Mycobacterium	K6	60813	100	66.3
DarthP	MF140406	Mycobacterium	K6	61594	94	67.2
Hammy	KY087993	Mycobacterium	K6	61812	94	67.2
Krueger	MF324914	Mycobacterium	K6	60321	100	66.5
PhelpsODU	MF324909	Mycobacterium	K6	56580	90	66.1
Phrank	MF324912	Mycobacterium	K6	61109	101	66.2
SirPhilip	MF324911	Mycobacterium	K6	61882	97	66.7
Unicorn	MF324908	Mycobacterium	K6	61208	100	66.2
Aminay	MH509442	Mycobacterium	K7	60430	105	67.8
Appletree2	MF185726	Mycobacterium	L1	73808	125	58.9
JoeDirt	JF704108	Mycobacterium	L1	74914	126	58.8
LeBron	HM152763	Mycobacterium	L1	73453	123	58.8
UPIE	JF704113	Mycobacterium	L1	73784	122	58.8
Wamburgrxpress	MH744425	Mycobacterium	L1	74392	128	58.8
Archie	KT591489	Mycobacterium	L2	76271	128	58.7
BigCheese	MH834600	Mycobacterium	L2	72810	126	58.9
Breezona	KC691254	Mycobacterium	L2	76652	132	58.9
Crossroads	KF024731	Mycobacterium	L2	76129	133	58.9
Faith1	JF744988	Mycobacterium	L2	75960	129	58.9
Finemlucis	MF185728	Mycobacterium	L2	77031	131	58.9
Gardann	KX507361	Mycobacterium	L2	76012	126	58.9
GuuelaD	MF324910	Mycobacterium	L2	76315	129	58.9
LilDestine	MH779511	Mycobacterium	L2	75440	129	58.9
Loadrie	KU997639	Mycobacterium	L2	76492	133	59.0
Miley16	MF185730	Mycobacterium	L2	76653	132	58.9
MkaliMitinis3	KU234099	Mycobacterium	L2	75844	132	58.9
Nicholasp3	MF140422	Mycobacterium	L2	75822	130	58.9
Rumpelstiltskin	JN680858	Mycobacterium	L2	69279	110	58.9
Wilder	KX580962	Mycobacterium	L2	75806	130	58.9
Winky	KC661276	Mycobacterium	L2	76653	132	58.9
Zakai	KX580961	Mycobacterium	L2	76363	132	58.9
Clautastrophe	MF140405	Mycobacterium	L3	72055	122	59.2
Kingsolomon	MF140413	Mycobacterium	L3	69537	110	59.1
Krypton555	MF140414	Mycobacterium	L3	76075	130	59.3
Lolly9	KT281791	Mycobacterium	L3	75816	130	59.3
Lumos	KT372003	Mycobacterium	L3	75586	128	59.3
Nicholas	MF140421	Mycobacterium	L3	70413	112	59.2
Samty	MH727559	Mycobacterium	L3	75307	127	59.3

Snenia	KT281794	Mycobacterium	L3	75626	130	59.3
Whirlwind	KF024725	Mycobacterium	L3	76050	128	59.3
Bromden	MH576973	Mycobacterium	L4	70183	118	58.2
Bongo	JN699628	Mycobacterium	M1	80228	132	61.6
Bricole	KT591491	Mycobacterium	M1	81128	139	61.6
IPhane7	MH697587	Mycobacterium	M1	81036	137	61.6
PegLeg	KC900379	Mycobacterium	M1	80955	138	61.5
GardenSalsa	KY783914	Mycobacterium	M2	80309	142	60.9
GenevaB15	MF319184	Mycobacterium	M2	80123	144	60.8
MrMagoo	KY223999	Mycobacterium	M2	84303	155	60.8
Rey	JF937105	Mycobacterium	M2	83724	153	60.9
Aggie	MH697576	Mycobacterium	N	44333	67	66.5
Andies	MG099936	Mycobacterium	N	43779	65	66.2
Butters	KC576783	Mycobacterium	N	41491	66	65.8
Carcharodon	KM588359	Mycobacterium	N	43680	71	66.2
Charlie	JN256079	Mycobacterium	N	43036	68	66.3
Gex	MH697585	Mycobacterium	N	43697	71	66.3
MichelleMyBell	KF986246	Mycobacterium	N	42240	70	66.0
Panchino	KU935727	Mycobacterium	N	43516	66	65.9
PhancyPhin	KX756439	Mycobacterium	N	42454	69	66.1
Philonius	MG099948	Mycobacterium	N	43886	72	66.5
Phrann	KU935731	Mycobacterium	N	44872	67	66.3
Pipsqueaks	KU935730	Mycobacterium	N	43679	73	66.3
Redi	JN624851	Mycobacterium	N	42594	69	66.1
Rubeelu	MH399787	Mycobacterium	N	41491	66	65.8
Silvafighter	MH316570	Mycobacterium	N	43243	74	66.3
SkinnyPete	KU935729	Mycobacterium	N	43478	67	66.4
Tapioca	MH697593	Mycobacterium	N	44205	70	66.1
Xeno	KU935728	Mycobacterium	N	42395	69	66.8
Xerxes	KU935726	Mycobacterium	N	43698	72	66.3
Catdawg	KF017002	Mycobacterium	O	72108	128	65.4
Corndog	AY129335	Mycobacterium	O	69777	122	65.4
Dylan	KF024730	Mycobacterium	O	69815	121	65.4
Familton	MG099943	Mycobacterium	O	71807	129	65.4
Firecracker	JN698993	Mycobacterium	O	71341	128	65.5
JangDynasty	MG872838	Mycobacterium	O	70883	124	65.4
Ryadel	MH590592	Mycobacterium	O	72658	132	65.2
SchoolBus	MH020246	Mycobacterium	O	71651	128	65.4
YungJamal	KJ829260	Mycobacterium	O	70214	124	65.3
Zakhe101	KT281796	Mycobacterium	O	69653	123	65.5
Arib1	MF919492	Mycobacterium	P1	46732	78	67.5
Bartholomew	MF140399	Mycobacterium	P1	46484	77	67.2
BigNuz	JN412591	Mycobacterium	P1	48984	82	66.7
Bogie	MF133446	Mycobacterium	P1	48639	81	66.9
Brusacoram	KT347313	Mycobacterium	P1	47618	78	67.0
Donovan	KF841477	Mycobacterium	P1	47162	78	67.2
Fishburne	KC691256	Mycobacterium	P1	47109	77	67.3
Jebeks	JN572061	Mycobacterium	P1	45580	75	67.3
Ksquared	MF281061	Mycobacterium	P1	48699	80	67.1

Majeke	MF472894	Mycobacterium	P1	47612	81	67.4
Malithi	KP027200	Mycobacterium	P1	46869	79	67.1
Nazo	KX641262	Mycobacterium	P1	48870	83	66.8
Phayonce	KR080195	Mycobacterium	P1	49203	77	66.7
Shipwreck	KU985090	Mycobacterium	P1	48670	81	66.9
StevieRay	MF373843	Mycobacterium	P1	48815	81	66.9
Thespis	MG198785	Mycobacterium	P1	47618	78	67.0
Tortellini	KX648391	Mycobacterium	P2	49658	76	65.8
Xavia	MH230879	Mycobacterium	P3	49808	71	65.9
Amochick	MH697577	Mycobacterium	Q	54145	86	67.4
Evanesce	KT454972	Mycobacterium	Q	53746	85	67.4
Gancho	MH727549	Mycobacterium	Q	54125	86	67.5
Giles	EU203571	Mycobacterium	Q	53746	78	67.4
HH92	KJ538722	Mycobacterium	Q	53746	84	67.4
Kinbote	KT222940	Mycobacterium	Q	53746	84	67.4
LilHazelnut	MF919517	Mycobacterium	Q	53746	85	67.5
OBUpride	KT246485	Mycobacterium	Q	53790	84	67.5
Nilo	MH001447	Mycobacterium	R	71752	102	56.0
Papyrus	KF416342	Mycobacterium	R	70657	100	56.0
Send513	JF704112	Mycobacterium	R	71547	98	56.0
Weiss13	KT591076	Mycobacterium	R	71436	100	55.9
Zenon	KY969628	Mycobacterium	R	71726	102	56.0
Gattaca	KX159477	Mycobacterium	S	65237	115	63.3
LittleLaf	MH727553	Mycobacterium	S	64834	112	63.4
Marvin	JF704100	Mycobacterium	S	65100	107	63.4
MosMoris	KJ538721	Mycobacterium	S	65243	111	63.4
Tesla	MG757167	Mycobacterium	S	64625	111	63.5
VasuNzinga	MH727562	Mycobacterium	S	64911	113	63.4
Dori	JN698995	Mycobacterium	Singleton	64613	94	66.0
DS6A	JN698994	Mycobacterium	Singleton	60588	97	68.4
Kumao	MG009575	Mycobacterium	Singleton	70373	115	62.1
MooMoo	MH001449	Mycobacterium	Singleton	55178	97	62.0
Sparky	KM083128	Mycobacterium	Singleton	63334	91	65.2
Bernal13	KJ510413	Mycobacterium	T	42392	60	66.2
Mendokysei	MG925349	Mycobacterium	T	43511	65	66.4
Nairb	MH001451	Mycobacterium	T	42393	59	66.2
RonRayGun	KM591905	Mycobacterium	T	42596	61	66.1
ZenTime222	MF155936	Mycobacterium	T	43344	67	66.0
Madruga	KR997933	Mycobacterium	U	69377	106	50.4
Patience	JN412589	Mycobacterium	U	70506	110	50.3
Cosmo	KP027195	Mycobacterium	V	78229	148	56.8
EniyanLRS	KY385381	Mycobacterium	V	78536	137	56.9
Wildcat	DQ398052	Mycobacterium	V	78296	148	56.9
Jeon	MH001450	Mycobacterium	W	60908	85	67.6
Megabear	MH001446	Mycobacterium	W	60579	89	67.5
Taptic	KY130461	Mycobacterium	W	60973	92	67.4
Gaia	KJ567043	Mycobacterium	X	90460	179	56.8
Bipper	KU728633	Mycobacterium	Y	77832	135	67.3
32HC	KJ028219	Mycobacterium	Z	50781	86	65.7

Rem711	MG770216	Mycobacterium	Z	50832	85	66.2
Aquarius	MF919491	Propionibacterium	BU	30112	48	54.5
ATCC29399BC	JX262225	Propionibacterium	BU	29516	46	54.0
ATCC29399BT	JX262224	Propionibacterium	BU	29516	46	54.0
Attacne	KR337651	Propionibacterium	BU	28876	45	54.7
BruceLethal	KR347352	Propionibacterium	BU	29249	44	54.0
DrParker	MF919505	Propionibacterium	BU	29742	45	54.6
Enoki	KR347353	Propionibacterium	BU	29347	45	54.6
Keiki	KR337649	Propionibacterium	BU	29339	45	54.0
Kubed	KR337645	Propionibacterium	BU	29461	44	54.5
Lauchelly	KR337650	Propionibacterium	BU	29517	45	53.9
Leviosa	MF919515	Propionibacterium	BU	29451	44	53.7
LilBandit	MF919516	Propionibacterium	BU	29041	45	54.0
MEAK	MF919522	Propionibacterium	BU	29223	44	54.0
Moyashi	KR347354	Propionibacterium	BU	29254	44	54.6
MrAK	KR337643	Propionibacterium	BU	29726	46	54.4
Ouroboros	KR337654	Propionibacterium	BU	29506	45	53.9
P1.1	JX262223	Propionibacterium	BU	29348	45	54.4
P100.1	JX262222	Propionibacterium	BU	29612	47	54.1
P100A	JX262221	Propionibacterium	BU	29505	45	53.8
P100D	JX262220	Propionibacterium	BU	29506	47	53.8
P101A	JX262217	Propionibacterium	BU	29574	47	54.1
P104A	JX262218	Propionibacterium	BU	29371	45	54.0
P105	JX262219	Propionibacterium	BU	29202	45	54.2
P14.4	JX262216	Propionibacterium	BU	29729	47	54.1
P9.1	JX262215	Propionibacterium	BU	29214	45	54.1
PA6	DQ431235	Propionibacterium	BU	29739	44	54.0
PAD20	NC_015454	Propionibacterium	BU	29074	45	54.1
PAS50	NC_015453	Propionibacterium	BU	29017	46	54.0
PHL010M04	NC_022336	Propionibacterium	BU	29511	45	54.0
PHL037M02	NC_022339	Propionibacterium	BU	29443	45	53.8
PHL060L00	NC_022338	Propionibacterium	BU	29514	46	54.0
PHL067M10	NC_022335	Propionibacterium	BU	29377	45	54.3
PHL071N05	NC_022337	Propionibacterium	BU	29467	45	53.9
PHL111M01	NC_022342	Propionibacterium	BU	29140	45	54.3
PHL112N00	NC_022334	Propionibacterium	BU	29266	46	54.5
PHL113M01	NC_022341	Propionibacterium	BU	29200	44	54.1
PHL114L00	NC_022340	Propionibacterium	BU	29464	46	54.2
Pirate	KR337653	Propionibacterium	BU	29328	44	54.1
Procrass1	KR337644	Propionibacterium	BU	29347	45	54.0
QueenBey	KR347355	Propionibacterium	BU	29338	43	54.1
SKKY	KR337648	Propionibacterium	BU	29594	47	54.6
Solid	KR337647	Propionibacterium	BU	29440	45	53.9
Stormborn	KR337652	Propionibacterium	BU	29330	46	53.8
Supernova	MF919533	Propionibacterium	BU	29217	45	54.4
Wizzo	KR337646	Propionibacterium	BU	29463	44	54.4
Anatole	KX620748	Propionibacterium	BV	35284	55	64.4
B3	KX620749	Propionibacterium	BV	35948	57	64.4
E1	KX620752	Propionibacterium	BV	35209	55	64.4

B22	KX620750	Propionibacterium	BW	37219	61	65.5
Doucette	KX620751	Propionibacterium	BW	37429	61	65.6
E6	KX620753	Propionibacterium	BW	38067	60	65.2
G4	KX620754	Propionibacterium	BW	38555	69	65.7
PFR1	NC_031076	Propionibacterium	BX	38071	56	64.8
PFR2	NC_031108	Propionibacterium	BX	39640	58	65.0
B5	NC_003460	Propionibacterium	Singleton	5804	10	64.3
Alatin	MF324905	Rhodococcus	CA	46673	65	58.7
Alpacados	MH271291	Rhodococcus	CA	46493	66	58.9
AngryOrchard	KY549153	Rhodococcus	CA	46597	65	58.8
AppleCloud	MF324903	Rhodococcus	CA	46389	64	58.7
BobbyDazzler	KY549154	Rhodococcus	CA	46641	65	58.8
Bonanza	MF537628	Rhodococcus	CA	46932	66	58.8
Bradshaw	MH271293	Rhodococcus	CA	46606	65	58.6
Bryce	MH271294	Rhodococcus	CA	46347	65	58.8
CosmicSans	KT372002	Rhodococcus	CA	46596	66	58.5
Erik	MH271297	Rhodococcus	CA	46429	66	58.5
Gollum	MH271299	Rhodococcus	CA	46535	66	58.6
Harlequin	KX611788	Rhodococcus	CA	46383	66	58.8
Hiro	MF324898	Rhodococcus	CA	46854	65	58.7
Jester	MF373842	Rhodococcus	CA	46314	65	58.7
Krishelle	MF324902	Rhodococcus	CA	46985	67	58.5
Lillie	KT990218	Rhodococcus	CA	46596	66	58.6
Naiad	MF324901	Rhodococcus	CA	46619	65	58.6
Nancinator	MH271306	Rhodococcus	CA	45936	65	58.6
Natosaleda	KX550082	Rhodococcus	CA	46527	65	58.6
Partridge	KX712237	Rhodococcus	CA	46962	66	58.8
Phrankenstein	MH271309	Rhodococcus	CA	46540	66	58.6
Rasputin	MH271311	Rhodococcus	CA	46568	66	58.8
RER2	JN116827	Rhodococcus	CA	46586	66	58.6
RexFury	MF324904	Rhodococcus	CA	46627	65	58.6
RGL3	JN116826	Rhodococcus	CA	48072	66	62.7
Rhodalysa	KT375356	Rhodococcus	CA	46596	66	58.5
Shuman	MH316569	Rhodococcus	CA	46544	67	58.6
StCroix	MF324900	Rhodococcus	CA	46619	65	58.6
Swann	MH271314	Rhodococcus	CA	46596	66	58.6
Takoda	MH271315	Rhodococcus	CA	46807	67	58.7
TWAMP	KT959213	Rhodococcus	CA	46596	66	58.5
UhSalsa	MH271319	Rhodococcus	CA	46539	66	58.6
Yogi	KX712236	Rhodococcus	CA	46930	66	58.8
Yoncess	MF189179	Rhodococcus	CA	46353	65	58.8
Grayson	MH153812	Rhodococcus	CB	131801	275	41.2
Peregrin	MH153807	Rhodococcus	CB	133006	273	41.4
Weasels2	KX774321	Rhodococcus	CB	134973	283	41.3
Pepy6	NC_023735	Rhodococcus	CC	76797	107	53.4
Poco6	NC_023694	Rhodococcus	CC	78064	107	53.3
ChewyVIII	KX557288	Rhodococcus	Singleton	69165	95	61.8
DocB7	NC_023706	Rhodococcus	Singleton	75772	105	56.7
E3	HM114277	Rhodococcus	Singleton	142563	220	67.5

Finch	MG962366	Rhodococcus	Singleton	138896	228	63.1
Jace	MH153804	Rhodococcus	Singleton	53912	93	67.0
Pine5	NC_023722	Rhodococcus	Singleton	59231	84	67.1
REQ1	NC_016655	Rhodococcus	Singleton	51342	85	66.3
REQ2	NC_016652	Rhodococcus	Singleton	49330	82	65.4
REQ3	NC_016654	Rhodococcus	Singleton	39474	60	65.9
RRH1	NC_016651	Rhodococcus	Singleton	14270	20	68.4
Trina	MF668286	Rhodococcus	Singleton	139262	252	44.7
SPB78	N/A	Streptomyces	BA	51282	77	71.2
Sros11	N/A	Streptomyces	BA	48399	77	70.3
VWB	AY320035	Streptomyces	BA	49220	74	71.1
phiBT1	AJ550940	Streptomyces	BB1	41831	55	62.8
phiC31	AJ006589	Streptomyces	BB1	41491	53	63.6
TG1	JX182372	Streptomyces	BB1	40474	54	64.6
Mojorita	KY092482	Streptomyces	BC1	38496	56	72.5
Picard	KY092480	Streptomyces	BC1	39522	56	72.6
Shyg	N/A	Streptomyces	BC1	39945	58	70.0
SV1	JX182371	Streptomyces	BC1	37612	55	72.7
ToastyFinz	KY676784	Streptomyces	BC1	39693	52	72.5
Darolandstone	MH825699	Streptomyces	BC2	40725	55	72.3
Raleigh	KY092484	Streptomyces	BC2	40785	53	71.8
Bioscum	KY092483	Streptomyces	BC3	37830	54	72.3
Ididsumtinwong	KY092479	Streptomyces	BC3	37817	55	72.4
PapayaSalad	KY092481	Streptomyces	BC3	38411	54	72.6
Aaronocolus	KT124227	Streptomyces	BD1	49562	73	66.2
BeardedLady	MF541403	Streptomyces	BD1	49941	74	66.2
Brataylor	KX507345	Streptomyces	BD1	51067	77	65.7
BryanRecycles	MF541404	Streptomyces	BD1	50066	75	65.9
Caliburn	KT152029	Streptomyces	BD1	49949	72	66.2
Celeste	MF541405	Streptomyces	BD1	50536	76	65.8
Danzina	KT124228	Streptomyces	BD1	50773	76	65.7
Dattran	MF541406	Streptomyces	BD1	50976	77	65.8
Eddasa	MH171096	Streptomyces	BD1	50605	76	65.9
Esperer	MF541407	Streptomyces	BD1	49908	74	66.2
Goby	MH171097	Streptomyces	BD1	51393	76	65.8
Godpower	KX507344	Streptomyces	BD1	50701	76	65.7
Hydra	KT124229	Streptomyces	BD1	50727	76	66.2
Izzy	KT184390	Streptomyces	BD1	50113	75	65.9
Jash	MF541408	Streptomyces	BD1	50066	75	65.9
Lannister	KT184391	Streptomyces	BD1	50165	73	65.7
Lika	KC700556	Streptomyces	BD1	51252	75	65.8
Lorelei	KX507343	Streptomyces	BD1	50558	75	65.8
Maneekul	MH171095	Streptomyces	BD1	51612	73	65.7
Nabi	MH171094	Streptomyces	BD1	51127	76	65.8
Nanodon	KX344445	Streptomyces	BD1	50082	75	65.7
Oliynyk	MF541409	Streptomyces	BD1	49976	75	65.9
Ozzie	MF541410	Streptomyces	BD1	49961	73	66.2
OzzyJ	MG757163	Streptomyces	BD1	51464	75	65.7
Rana	MH171093	Streptomyces	BD1	50980	76	65.8

Sujidade	KC700557	Streptomyces	BD1	51552	77	65.7
Toma	MH171098	Streptomyces	BD1	51396	76	65.8
Zemlya	KC700558	Streptomyces	BD1	51077	76	65.7
Amethyst	MF766044	Streptomyces	BD2	49372	79	67.2
Daudau	MF766045	Streptomyces	BD2	50602	83	67.1
Diane	MF766046	Streptomyces	BD2	50483	79	66.7
ELB20	JX262376	Streptomyces	BD2	51160	81	67.0
Haizum	MH590601	Streptomyces	BD2	50660	81	66.8
Hank144	MH669004	Streptomyces	BD2	50547	78	66.3
Omar	MG593802	Streptomyces	BD2	49299	80	67.3
Paedore	MH001460	Streptomyces	BD2	52743	84	67.0
R4	JX182370	Streptomyces	BD2	51071	86	67.0
SqueakyClean	MF766047	Streptomyces	BD2	50837	78	67.0
Tefunt	MF766048	Streptomyces	BD2	50574	81	66.8
Thestral	MH651190	Streptomyces	BD2	52628	82	67.7
TrvxScott	MH669016	Streptomyces	BD2	52600	81	67.8
Alsaber	MG298964	Streptomyces	BD3	48803	76	65.9
Amela	KT186228	Streptomyces	BD3	49452	75	65.6
phiCAM	JX889246	Streptomyces	BD3	50348	72	65.6
Verse	KT186229	Streptomyces	BD3	49483	75	65.6
Yosif	MH248947	Streptomyces	BD3	50129	78	66.1
phiHau3	JX182369	Streptomyces	BD4	50255	77	67.8
StrepC	N/A	Streptomyces	BD5	51580	77	67.4
Jay2Jay	KM652554	Streptomyces	BE1	133531	235	49.5
Mildred21	MF155946	Streptomyces	BE1	131976	234	49.5
NootNoot	MF347636	Streptomyces	BE1	131086	221	50.2
Paradiddles	MF347637	Streptomyces	BE1	133486	216	50.1
Peebs	MF347638	Streptomyces	BE1	133047	226	50.1
Samisti12	MF347639	Streptomyces	BE1	133710	226	49.9
Sushi23	MF358542	Streptomyces	BE1	133917	229	50.0
Warpy	MF358541	Streptomyces	BE1	132996	233	49.6
Karimac	MH590599	Streptomyces	BE2	131909	241	49.4
LukeCage	MH590597	Streptomyces	BE2	133195	248	49.0
Starbow	MH576964	Streptomyces	BE2	131427	238	49.5
StarPlatinum	MH576965	Streptomyces	BE2	133886	251	49.5
Wofford	MH576968	Streptomyces	BE2	133007	236	47.7
Yaboi	MH727564	Streptomyces	BE2	131250	242	49.3
FlowerPower	MH155868	Streptomyces	BF	46133	66	60.7
HaugeAnator	MG663582	Streptomyces	BF	46135	64	59.6
Immanuel3	MG518520	Streptomyces	BF	46094	62	59.6
Manuel	MG518519	Streptomyces	BF	45177	63	60.1
Percastrophe	MG663583	Streptomyces	BF	45999	64	59.7
Romero	MG663584	Streptomyces	BF	46079	64	59.7
ToriToki	MG663585	Streptomyces	BF	46077	64	59.7
WRightOn	MG515223	Streptomyces	BF	45221	64	60.3
ZooBear	MG663586	Streptomyces	BF	46135	64	59.7
Abt2graduatex2	MF975638	Streptomyces	BG	57385	71	69.2
BabyGotBac	KY365739	Streptomyces	BG	57165	72	69.2
BayC	MH178381	Streptomyces	BG	57243	71	69.2

Maih	KU189325	Streptomyces	BG	57256	70	69.3
Salete	MH178382	Streptomyces	BG	57243	71	69.2
TP1604	KP876466	Streptomyces	BG	57168	71	69.2
Xkcd426	KU530220	Streptomyces	BG	64477	78	68.8
YDN12	KP876465	Streptomyces	BG	56528	70	69.2
Crosby	MH536815	Streptomyces	BH	54036	82	68.3
Henoccus	MH229862	Streptomyces	BH	55137	82	68.2
JackieB	MH229863	Streptomyces	BH	54912	82	68.2
LazerLemon	MH229865	Streptomyces	BH	54798	81	68.1
Microdon	MH825706	Streptomyces	BH	53356	79	68.6
UNTPL	MH229864	Streptomyces	BH	54495	81	68.3
Bing	MG757154	Streptomyces	BI1	56341	87	59.4
DrGrey	MF467948	Streptomyces	BI1	56076	82	59.5
OlympicHelado	KX670789	Streptomyces	BI1	56189	88	59.5
Rima	KX670790	Streptomyces	BI1	56168	87	59.6
Spectropatronm	MF467949	Streptomyces	BI1	55707	85	59.5
HotFries	MH155869	Streptomyces	BI2	43699	57	60.9
Moozy	MH155872	Streptomyces	BI2	43545	58	61.2
RavenPuff	MH155878	Streptomyces	BI2	43709	59	60.9
Scap1	MF975637	Streptomyces	BI2	43060	56	60.9
LibertyBell	MH669006	Streptomyces	BI3	52733	79	59.1
Rainydai	MH155877	Streptomyces	BI4	57623	91	58.1
SendItCS	MH155880	Streptomyces	BI4	55993	86	58.2
phiSASD1	GQ379227	Streptomyces	BJ	37068	44	66.3
Annadreamy	MH536811	Streptomyces	BK1	125726	229	47.6
Blueeyedbeauty	MH536814	Streptomyces	BK1	130473	240	47.9
Comrade	MH651172	Streptomyces	BK1	129015	229	47.1
SparkleGoddess	MH590589	Streptomyces	BK1	129742	232	47.1
BillNye	MG757153	Streptomyces	BK2	127084	217	52.8
AbbeyMikolon	MG593800	Streptomyces	BL	42551	59	66.8
Nesbitt	MH001457	Streptomyces	BL	42446	58	66.8
Rowa	MG593803	Streptomyces	BL	42890	62	61.2
JustBecause	MH744418	Streptomyces	BM	184281	325	66.6
Satis	MH576962	Streptomyces	BM	186702	325	66.7
Wentworth	MH019216	Streptomyces	BN	68260	103	64.1
Yara	MH019215	Streptomyces	BN	68671	105	63.9
Attoomi	MG593801	Streptomyces	Singleton	41872	53	69.7
Chymera	KU958700	Streptomyces	Singleton	34742	55	71.4
Ibantik	MH155870	Streptomyces	Singleton	56362	105	57.4
Kromp	MH744420	Streptomyces	Singleton	58268	95	71.4
mu16	N/A	Streptomyces	Singleton	38196	56	71.2
phiSAV	N/A	Streptomyces	Singleton	41529	61	67.2
pZL12	N/A	Streptomyces	Singleton	90435	112	69.5
TJE1	NC_019930	Tetrasphaera	Singleton	49219	66	57.7
TPA2	NC_015210	Tsukamurella	Singleton	61440	78	69.6
TPA4	KR053196	Tsukamurella	Singleton	56212	84	70.5

[1]Primary phage identifier at PhagesDB.org
[2]Genbank or RefSeq accession, if available
[3]Cluster or Subcluster designation at PhagesDB.org

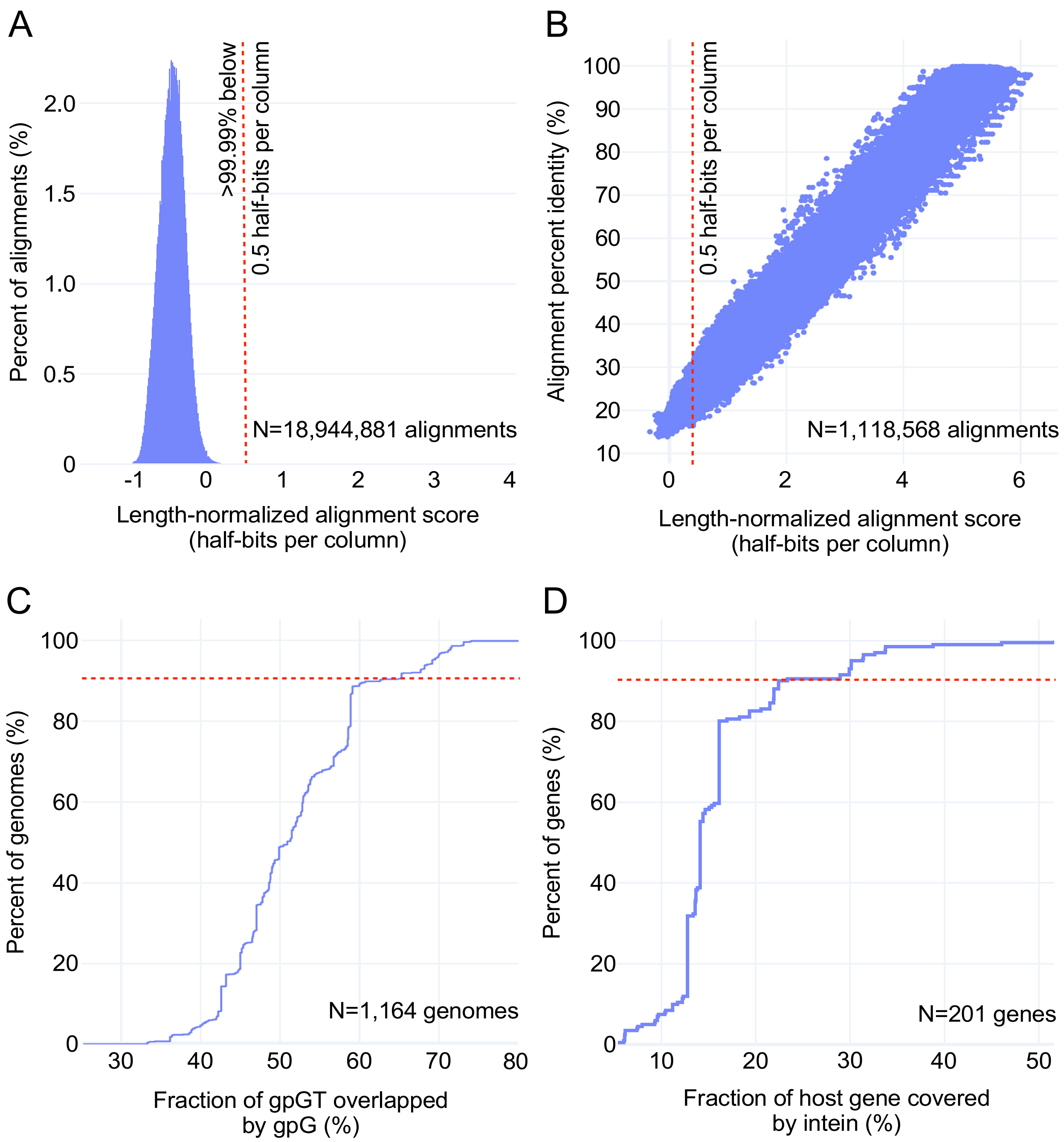
A
Percent of alignments (%)
2.0
1.5
1.0
0.5
0
-1
0
1
2
3
4
>99.99% below
0.5 half-bits per column
N=18,944,881 alignments
Length-normalized alignment score
(half-bits per column)
B
Alignment percent identity (%)
100
90
80
70
60
50
40
30
20
10
0
2
4
6
0.5 half-bits per column
N=1,118,568 alignments
Length-normalized alignment score
(half-bits per column)
C
Percent of genomes (%)
100
80
60
40
20
0
30
40
50
60
70
80
N=1,164 genomes
Fraction of gpGT overlapped
by gpG (%)
D
Percent of genes (%)
100
80
60
40
20
0
10
20
30
40
50
N=201 genes
Fraction of host gene covered
by intein (%)

Figure S1
